# Novel Aloe-Emodin Derivatives as Potential Anticancer Agents: Synthesis, Characterization and Cytotoxic Activity

**DOI:** 10.3390/molecules31101676

**Published:** 2026-05-15

**Authors:** Jeltzlin Semerel, Shuhe Zheng, Haoyue Hu, Yuyu Fang, Nigel John, Pedro Fardim, Wim Dehaen

**Affiliations:** 1 SISSTEM Program, Faculty of Arts and Science, University of Aruba, J. Irausquinplein 4, Oranjestad, Aruba; jeltzlin.semerel@ua.aw (J.S.); nigel.john@ua.aw (N.J.); 2 Institut de Science et D’ingénierie Supramoléculaires, University of Strasbourg, 8 Allée Gaspard Monge, 67000 Strasbourg, France; shuhe.zheng@ics-cnrs.unistra.fr; 3 School of Pharmacy, Chengdu University of Traditional Chinese Medicine, Chengdu 611137, China; huhaoyue@stu.cdutcm.edu.cn (H.H.); yyfang@cdutcm.edu.cn (Y.F.); 4 Department of Chemical Engineering, KU Leuven, Celestijnenlaan 200F, B-3001 Leuven, Belgium; pedro.fardim@kuleuven.be; 5 Department of Chemistry, KU Leuven, Celestijnenlaan 200F, B-3001 Leuven, Belgium

**Keywords:** anthra[1,2-*b*]furan, cytotoxic activity, aloe-emodin, Castro–Stephens coupling

## Abstract

The fusion of heterocycles onto an anthraquinone scaffold represents a promising strategy to optimize anticancer activity. This study has the aim to synthesize and characterize novel anthra[1,2-*b*]furan compounds based on the natural product aloe-emodin. Six novel anthra[1,2-*b*]furans bearing phenyl, *n*-hexane, and methoxy carbonyl substituents were synthesized starting from aloe-emodin. The synthetic route employed involved acetyl protection of aloe-emodin, electrophilic aromatic halogenation, subsequent Castro–Stephens coupling, spontaneous intramolecular cyclization, and deprotection of hydroxyl groups. These newly synthesized compounds were evaluated for their cytotoxic activity against various cancer cell lines, including lung adenocarcinoma (A5492), colorectal carcinoma (HCT116), hepatocellular carcinoma (HepG2), ovarian cancer (Skov3), and breast cancer (MCF-7), using the CCK8 assay. The anthra[1,2-*b*]furan derivative **10c**, which contains a methoxy carbonyl group, demonstrated excellent potency against lung (A549) and breast (MCF-7) cancer cell lines, with IC_50_ values of 0.49 and 2.91 µM, respectively. This preliminary cytotoxic finding shows compound **10c** as a promising hit for further investigations towards a promising lead compound.

## 1. Introduction

**Figure 1:**
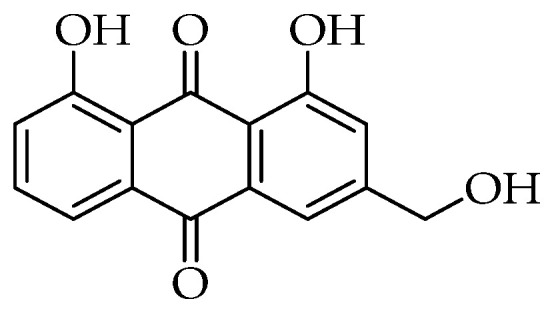
Aloe-emodin.

Aloe-emodin (Figure 1) is a naturally occurring anthraquinone that is widely found in plants such as *Cassia occidentalis*, *Polygonum multiflorum* Thunb, *Rheum palmatum*, and *Aloe vera* [1]. Aloe-emodin has been an attractive candidate for drug discovery over the years due to its broad range of biological applications as anticancer [2,3], anti-inflammation [4], antimicrobial [5], antioxidant [6], and photodynamic therapy [7,8] agents. In cancer research, aloe-emodin has shown excellent activity against a wide variety of cancer cell lines, e.g., bladder [9], breast [10], cervical [11], colon [12], gastric [13], leukemia [14], liver [15], lung [16,17], nasopharyngeal [18,19], neuroectodermal tumors [20], oral [21], ovarian [22], prostate [23], and tongue [24] cancer cells. The potent effects of aloe-emodin likely stem from its planar and rigid structure, which allows it to intercalate between the DNA base pairs within the double helix [25,26,27], thereby inducing irreversible cellular damage [28,29,30]. Although aloe-emodin shows promising activity with wide application in cancer research, it possesses challenges for drug development. Its application to a wide variety of cancer cell types has been reported to occur via five primary signaling pathways, e.g., induction of cell cycle arrest and apoptosis, inhibition of metastasis and angiogenesis, regulation of autophagy, overcoming drug resistance, and regulating tumor microenvironment [31]. Its ability to target multiple signaling pathways results in poor selectivity and specificity, which are needed for effective drug targeting. This also leads to challenges in understanding its specific biological effects in the human body and the emergence of side effects due to long-term exposure [31]. In toxicity studies, aloe-emodin has been shown to be phototoxic, hepatotoxic, nephrotoxic, and genotoxic, which are all of concern for future clinical applications [1,3]. Lastly, aloe-emodin has poor water solubility, low intestinal absorption, and poor bioavailability [32,33].

**Figure 2:**
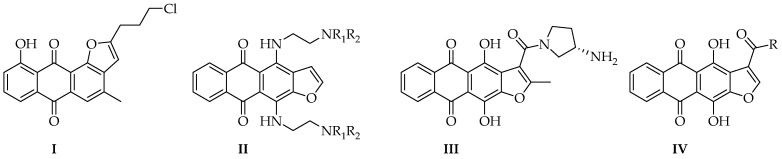
Promising anticancer agents of anthraquinone with five-membered ring fusions, (**I**) anthra[1,2-*b*]furan with chloropropyl substituent, (**II**) 4,11-diaminoanthra[2,3-*b*]furan-5,10-diones, (**III**) anthra[2,3-*b*]furan with (*S*)-3-aminopyrrolidine substituent and (**IV**) anthra[2,3-*b*]furan with carboxamide spacer group.

Significant work has been dedicated in the last few decades (2003–2025) to the study of the biological properties of aloe-emodin that has been modified via the phenolic and benzylic hydroxyl groups [34]. For example, Kumar et al. [35] synthesized aloe-emodin derivatives bearing pyrazole substituents that showed improved potency against breast, liver, and skin cancer cells. In another study, aloe-emodin was modified at the benzylic hydroxyl position to incorporate furan substituents that showed high cytotoxic activity against oral cancer cell through CLK kinase inhibition [36]. Aromatic carbon functionalization of aloe-emodin for improved biological potency remains an unexplored research area. Several anthraquinones bearing ring structures such as furan moieties have shown promising anticancer activity (Figure 2). For example, anthra[1,2-*b*]furan **I** showed the highest growth inhibition against multiple cancer cells lines with IC_50_ values ranging from 0.38 to 1.9 µM [37]. In another study, anthra[2,3-*b*]furan derivatives **II** showed good antiproliferative activity against lymphocytic leukemia (L1210), T lymphoblast (CEM), cervical cancer (HeLa), and colon cancer (HCT116) cell lines with IC_50_ value ranging from 0.10 to 0.55 µM [38]. On the other hand, anthra[2,3-b]furans **III** with carboxamides as a spacer group showed good inhibition of topoisomerases (1 & 2) and protein kinases, while exhibiting potent activity against drug-resistant tumor cells [39]. Further structural substitutions at the carboxamide spacer group led to the production of anthra[2,3-b]furans **IV**. The analog **IV** containing (S)-3-aminopyrrolidine and 3-aminopiperidine substituents showed the highest antiproliferative potency against leukemia (L210 & K562), T lymphoblast (CEM), cervical cancer (HeLa), and colon cancer (HCT116) with IC_50_ values in the range of 0.6–3.4 µM. These derivatives also showed inhibition of topoisomerase 1 and were able to bypass drug resistance in gastric cancer cells leading to apoptosis despite p53 deficiency [40]. Five-membered ring fusion on the anthraquinone core shows promising results for anticancer research. This type of fusion maintains the planarity and rigidity of the anthraquinone structure, while also providing opportunities for modifications of its physical properties.

In this study, we report a series of anthra[1,2-*b*]furan derivatives synthesized from aloe-emodin via a four-step route, including acetyl protection of aloe-emodin, selective halogenation, Castro–Stephens coupling, spontaneous intramolecular cyclization, and deprotection of the hydroxyl groups. A preliminary screening was performed to analyze the cytotoxic activity of the anthra[1,2-*b*]furan derivatives against five cancer cell lines: lung adenocarcinoma (A5492), colorectal carcinoma (HCT116), hepatocellular carcinoma (HepG2), ovarian cancer (Skov3), and breast cancer (MCF-7).

## 2. Results and Discussion

### 2.1. Chemistry

**Scheme 1:**
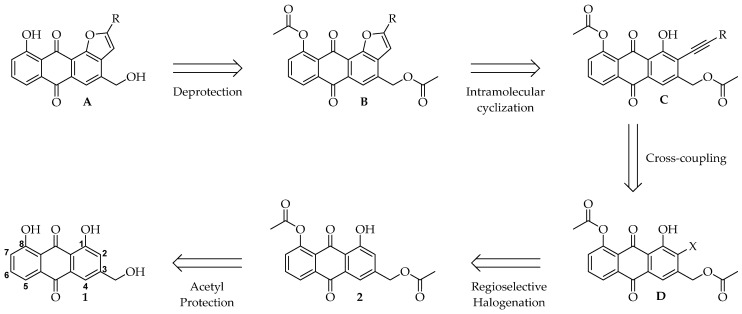
Retrosynthetic analysis for anthra[1,2-*b*]furan synthesis, (**A**) anthra[1,2-*b*]furan, (**B**) acetyl protected anthra[1,2-*b*]furan, (**C**) alkyne intermediate, (**D**) acetyl protected and halogenated anthraquinone, (**2**) acetyl protected aloe-emodin and (**1**) aloe-emodin.

The strategy to incorporate a furan group at the C2 position of aloe-emodin **1** to produce anthra[1,2-*b*]furan **A** derivatives is shown in Scheme 1. This strategy includes acetyl protection of hydroxyl groups, halogenation, cross-coupling, intramolecular cyclization, and deprotection of the hydroxyl groups. Our preliminary results indicated that halogenation of aloe-emodin **1** yielded multiple substituted products that could not be separated. Horvat et al. reported similar findings for the halogenation of emodin, which also produced multiple substituted products [41]. Therefore, direct halogenation of aloe-emodin **1** to afford intermediate **D** was not considered an option. Bringmann et al. [42] reported that bromine could be introduced to the C2 position of aloe-emodin **1** by brominating the acetylated intermediate **2**. Thus, selective protection of the 8-OH group with an acetyl group leaves the 1-OH group open to act as an ortho director for electrophilic aromatic substitution [43]. The strategy was adapted to produce intermediate **C** by applying Sonogashira cross-coupling to acetylated intermediate **D** (X = Br).

**Scheme 2:**
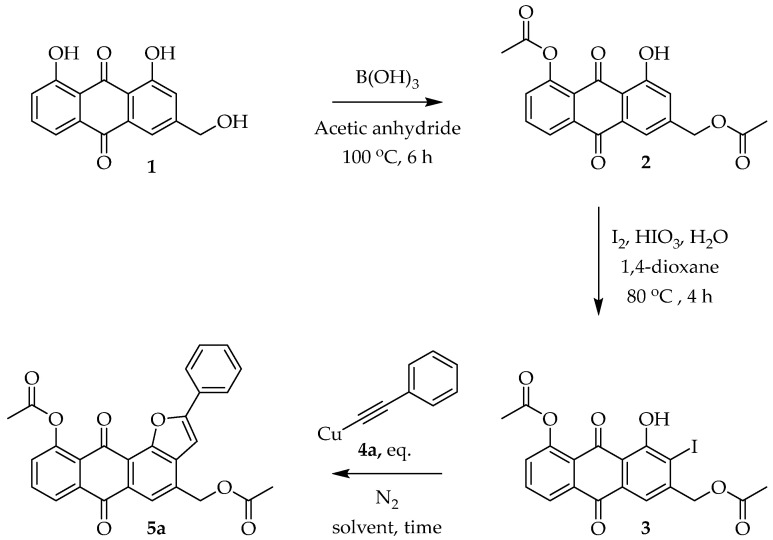
Synthesis of anthracenone furan **5a**, (**1**) aloe-emodin, (**2**) acetyl protected aloe-emodin, (**3**) aloe-emodin iodide (**4a**) (phenylethynyl) copper and (**5a**) acetyl protected anthra[1,2-*b*]furan.

The introduction of an alkyne substituent could not be achieved using the acetylated intermediate **D** (X = Br), despite multiple attempts with different acetylenes, palladium sources, temperatures, and solvents. The size of the palladium source could have hindered the cross-coupling between the alkynyl moiety and the 2-position of the anthraquinone due to steric crowding at that position. Castro–Stephens cross-coupling was considered as a potential substitution for the Sonogashira reaction, due to the use of the less sterically hindered organometallic copper(I) reagent. The Castro–Stephens strategy also did not lead to the cross-coupling between the bromine and an alkyne substituent which was likely due to the strong bond between the aryl group and the bromine. To facilitate cross-coupling, replacement of the bromine on the acetylated intermediate **D** with a more reactive iodine atom was considered. Iodination of acetylated intermediate **2** with iodine (I_2_) and iodic acid (HIO_3_) afforded aloe-emodin iodide **3** in 78% yield (Scheme 2). For the synthesis of anthra[1,2-*b*]furan **A**, a Castro–Stephens cross-coupling reaction previously reported by Mzhelskaya and Rixson et al. [37,43] was considered. Both studies describe methods for synthesizing anthra[1,2-*b*]furan derivatives in good yield using iodoanthracenes and acetylenes. Therefore, similar methods were applied to synthesize anthra[1,2-*b*]furan **5a** from the aloe-emodin iodide **3** (Table 1).

The Castro–Stephens coupling of aloe-emodin iodide **3** yielded 20% anthra[1,2-*b*]furan **5a**, when (phenylethynyl)copper **4a** in DMF was used under reflux for 1.5 h (Table 1, **Entry 1**). The yield of **5a** was only slightly improved to 21% despite the increase in reaction time from 1.5 to 4 h (Table 1, **Entry 2**). This could have happened due to the decomposition of intermediate **3** at the high reaction temperature of 150 °C. However, reducing the reaction temperature to 110 °C and extending the reaction time to 48 h further decreased the yield to 10% (Table 1, **Entry 3**), likely due to the poor solubility of (phenylethynyl)copper **4a** at low temperatures. Further optimization was accomplished by increasing the amount of (phenylethynyl)copper **4a** from 1.5 to 3.0 eq. and reacting at 150 °C for 1.5 h, which improved the yield to 30% (Table 1, **Entry 4**). The yield was further improved to 47% by running the reaction under microwave-irradiation at 150 °C for 30 min (Table 1, **Entry 5**). Changes in the solvent type led to a decrease in the yield (Table 1, **Entry 6–7**). Another strategy employed was to improve the solubility of (phenylethynyl)copper **4a** by adding dimethylethylenediamine (DMEDA). Rixson et al. [37] reported a yield of up to 64% for the synthesis of anthra[1,2-*b*]furan using (phenylethynyl)copper **4a** in toluene with DMEDA as the base at 90 °C for 18 h. However, this method did not lead to production of the desired product **5a** even with an extension of the reaction time to 72 h (Table 1, **Entry 8**).

**Scheme 3:**
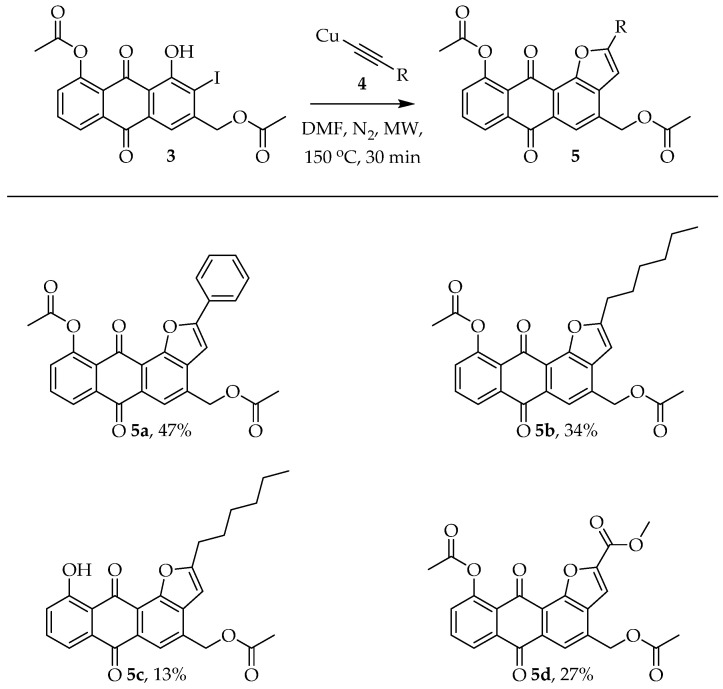
Synthesis of anthra[1,2-*b*]furan derivatives starting from the C2 position.

The optimal reaction conditions chosen for the synthesis of **5a** were 1.0 eq. aloe-emodin iodide **3**, 3.0 eq. cuprous acetylide in DMF under nitrogen with microwave-assistance at 150 °C for 30 min. Anthra[1,2-*b*]furan derivatives with *n*-hexyl and methoxycarbonyl substituents starting from aloe-emodin iodide **3** were synthesized with the established optimal conditions (Scheme 3). Protected anthra[1,2-*b*]furan **5b** and mono-protected anthra[1,2-*b*]furan **5c** containing the *n*-hexyl moiety were produced in 34% and 13% yield, respectively. On the other hand, the protected anthra[1,2-*b*]furan **5d** bearing the methoxy carbonyl substituent was synthesized in 27% yield.

**Scheme 4:**
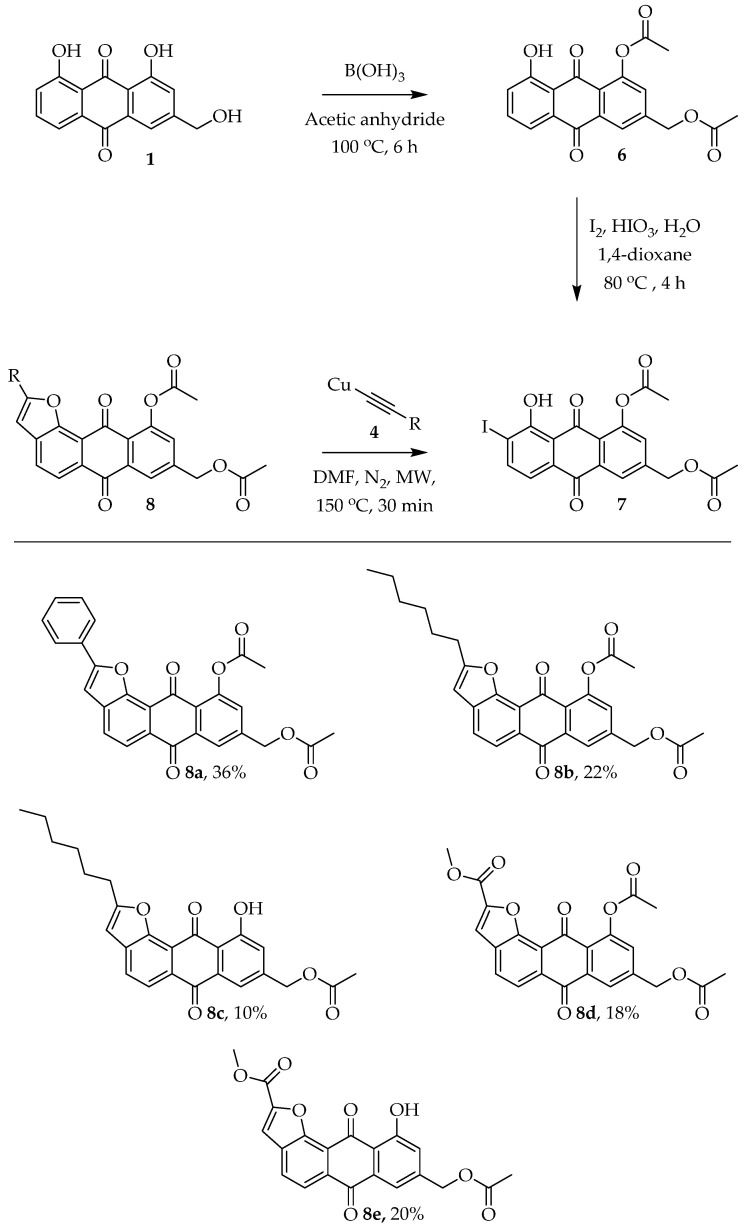
Synthesis of anthra[1,2-*b*]furan derivatives starting from the C7 position.

Many studies focus on synthesizing ring-fused anthraquinones through the C1 position, but there has not been any documentation on C7 modifications or their relevance for improved cytotoxic activity. Therefore, anthra[1,2-*b*]furans **8** were synthesized starting from the acetylated intermediate **6** (Scheme 4). The acetylated intermediate **6** is an isomer that is produced together with compound **2** during the acetylation reaction. Purification of acetylated intermediate **6** proved challenging despite the use of multiple solvent systems as chromatography eluent, including mixtures containing chloroform, petroleum ether, toluene, dichloromethane. Ultimately, partial purification of **6** was accomplished by repeated crystallization with chloroform–petroleum ether (1:1) followed by toluene. Iodination of acetylated intermediate **6** yielded aloe-emodin iodide **7** in 77% yield, undergoing similar ortho directing principle as compound **2**. The established optimal conditions were further applied to synthesize anthra[1,2-*b*]furans **8a**–**e** in low to fair 10–36% yield.

**Figure 3:**
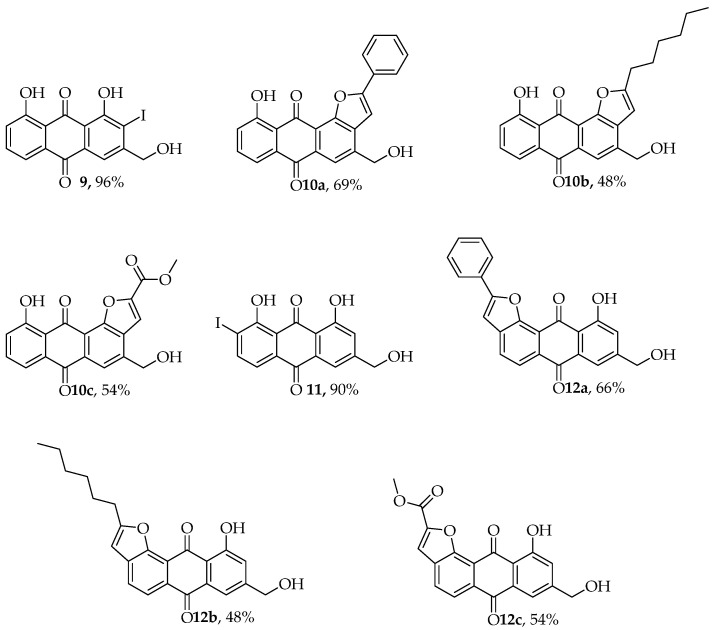
Novel anthra[1,2-*b*]furan derivatives.

The main challenge encountered was the purification step. The Castro–Stephens cross-coupling reaction led to multiple side products including partial deprotection, which were not easily separated from the desired product due to close polarities and poor solubility in multiple organic solvents systems. Anthra[1,2-*b*]furans **8d** and **8e** proved to be the most challenging to characterize due to purification issues arising due to poor solubility, multiple side products and close polarity that afforded low yields. Therefore, an alternative strategy was employed, to confirm the synthesis of anthra[1,2-*b*]furans **8d** and **8e**. The alternative strategy was to immediately deprotect the hydroxyl groups after Castro–Stephens cross-coupling without prior purification to afford the anthra[1,2-*b*]furan **12c** (Figure 3). The crude product obtained from the cross-coupling, containing **8d** and **8e**, was added to methanolic HCl under reflux overnight to produce the anthra[1,2-*b*]furan **12c** in 54% yield. The same strategy was employed for derivatives **10a**–**c** and **12a**–**b** to investigate whether higher overall yields could be achieved. As a result, the purification efficiency via column chromatography was significantly improved, and an increase in overall yield of 48–69% was observed for both **10a**–**c** and **12a**–**b** derivatives (Figure 3).

### 2.2. Cytotoxic Evaluation

The cytotoxic activity of the anthra[1,2-*b*]furans against lung (A549), colon (HCT116), liver (HEPG2), ovarian (Skov3), and breast (MCF-7) cancer cell lines was compared to aloe-emodin **1** and its iodinated derivatives **9** and **11** (Table 2). Anthra[1,2-*b*]furan derivative **10c** showed the highest cytotoxic activity against all cancer cell lines compared to aloe-emodin **1** and other derivatives. The highest cytotoxic activity of **10c** was observed against A549 and MCF-7 with IC_50_ values of 0.49 and 2.91 µM, respectively. The preliminary analysis on the structure–activity relationship (SAR) shows that (1) the introduction of iodine at the C2 position improved potency, (2) there was no correlation between improved cytotoxicity and the positioning of the furan group to either the C2 or C7 position, and (3) C2-positioned anthra[1,2-*b*]furans with a methoxy carbonyl substituent were essential for improved cytotoxic activity against all cancer cell lines. Compound **9** containing an iodine at the C2 position showed improved cytotoxic activity, while iodination at C8 as for **11** led to a worsened potency compared to aloe-emodin **1**. Noticeably, the cytotoxicity improved from acetylated intermediate **2** to acetylated iodide intermediate **3** to the non-acetylated iodide intermediate **9**. However, the opposite was true for iodination at the C8 position, whereby the potency improved from non-acetylated iodide intermediate **11** to acetylated iodide intermediate **7** towards acetylated intermediate **6**. Further comparison between the acetylated and non-acetylated anthra[1,2-*b*]furans in the future could elucidate whether the 1-hydroxyl and 8-hydroxyl groups are essential for cytotoxicity. Multiple reports on anthraquinones have highlighted the importance of hydroxyl groups for hydrogen bonding with DNA and/or amino acid residues [27,44,45,46]. The positioning of the iodine seems to be important for improved cytotoxicity. In contrast, the position of the furan group at either the C2 or C7 position did not lead to clear cytotoxicity improvements. For instance, derivatives containing the methoxy carbonyl (**10c** and **12c**) group showed the highest cytotoxicity activity compared to the other anthra[1,2-*b*]furans. Compounds containing phenyl and *n*-hexyl groups showed less cytotoxicity compared to aloe-emodin **1**. These results indicate the importance of the methoxycarbonyl group for the cytotoxicity of anthra[1,2-*b*]furans **10c**. Anthra[1,2-*b*]furan **10a** showed the least potency compared to all tested derivatives, which could be due to its poor solubility in most organic solvents, including DMSO which was the solvent used for the cytotoxic evaluation. Poor solubility can lead to underestimation of cytotoxic activity due to compound precipitation [47].

## 3. Materials and Methods

### 3.1. General Section

Chemicals and solvents were purchased from commercial sources (Acros Organics (Geel, Belgium), BLD Pharmatech (Reinbek, Germany), Carl Roth, Fisher Scientific (Loughborough, Belgium), Fluorochem EU Limited (Hadfield, UK), or Merck Life Science (Darmstadt, Germany)) and used without further purification. The microwave-assisted reactions were performed using the CEM-Discover Microwave Synthesizer, a flexible single instrument. The reactions were performed in a 10 mL glass tube sealed with a Teflon septum. Thin-layer chromatography (TLC) was performed on silica gel (60 Å pore size) with a fluorescent indicator (254 nm). TLC visualization was performed with UV irradiation at 254 nm or 365 nm. Column chromatography was performed using 70–230 mesh silica gel 60 (Acros Organics, Geel, Belgium) as the stationary phase. Melting points were analyzed on purified derivatives using a Reichert Thermovar (Carrollton, TX, USA) instrument. High-field nuclear magnetic resonance (NMR) spectra were recorded on a Bruker Avance III HD 400 spectrometer with a Bruker AscendTM 400 magnet system (1H basic frequency of 400.17 MHz) and a 5 mm PABBO BB/19F-1H/D probe with z-gradients or on a Bruker Avance II+ 600 spectrometer with a Bruker 600 UltraShieldTM magnet system (1H basic frequency of 600.13 MHz) and a 5 mm PABBO BB-1H/D probe with z-gradients. ^13^C-detected experiments were ^1^H-decoupled using power-gated and inverse-gated broadband decoupling, respectively. All samples were dissolved in chloroform-*d* (CDCl_3_) or DMSO-*d_6_*. Data were recorded at room temperature using Bruker TopSpin 3.x.x (Bruker Avance III HD 400 and Bruker Avance II+ 600 spectrometers) and processed and analyzed using Bruker TopSpin 4.2.x. ^1^H data were calibrated using tetramethylsilane (TMS) as an internal calibration reference, while ^13^C data were calibrated using the deuterated solvents as internal calibration reference (for CDCl_3_, a 1:1:1 triplet at 77.16 ppm; and for DMSO-*d_6_*, a 1:3:6:7:6:3:1 septet at 39.52 ppm). The chemical shifts (δ) were expressed in parts per million (ppm). The following acronyms were used for multiplicity: singlet (s), doublet (d), triplet (t), quartet (q), multiplet (m). The prefix app. denotes the apparent multiplicity of a signal, indicating the general shape and form of the multiplet in the spectrum, even though this is not theoretically expected based on the molecular structure of the compound and/or some higher-order fine structure could be observed. The coupling constant (J) is reported in Hertz (Hz). High-resolution mass spectra (HRMS) with exact masses were obtained using a quadrupole orthogonal acceleration time-of-flight mass spectrometer (Synapt G2 HDMS, Waters, Milford, MA, USA). Samples were infused at 3 mL/min and spectra were obtained in positive ionization mode with a resolution of 15,000 FWHM (full width at half maximum) using leucine enkephalin as a lock mass. The synthesized compounds 2-12c are provided in the Supplementary Material.

### 3.2. Experimental Section

#### 3.2.1. General Protocol for Acetylation

The procedure by Alexander et al. [48] was used with minor modifications. Boric acid (1.0 g, 16 mmol) was added to acetic anhydride (20 mL, 0.36 mol) and stirred at 100 °C until complete dissolution. Thereafter, aloe-emodin (1.0 g, 3.8 mmol) was added to the mixture and reacted at 100 °C for 6 h. Conversion was monitored via TLC. The mixture was left to cool down to room temperature once full conversion was achieved. The mixture was poured into water (60 mL) and heated at 50 °C for 30 min for acetic anhydride hydrolysis to occur. The mixture was cooled after to room temperature, at which point a yellow solid precipitate developed. This precipitate was filtered via vacuum filtration, dried in a vacuum oven, and further purified via crystallization. The yellow solid was a mixture of compounds **2** and **6**. R_f_ = 0.9 (DCM/MeOH 9:1).

#### 3.2.2. General Protocol for Iodination

The procedure by Mzhelskaya et al. [43] was used with minor modifications. A mixture of iodine (375 mg, 3.0 mmol) and iodic acid (260 mg, 1.5 mmol) dissolved in water (3 mL) was added to a solution consisting of acetylated aloe-emodin dissolved in 1,4-dioxane (9 mL) and left to react at 80 °C for 4 h. Conversion was monitored via TLC. The mixture was left to cool down to room temperature once full conversion was achieved. The mixture was poured into water (50 mL), and the precipitate was filtered via vacuum filtration and recrystallized from toluene.

#### 3.2.3. General Protocol for Cuprous Acetylide Synthesis

The procedure from Rixson et al. [37] was used and adapted with minor modifications. A mixture of CuCl (200 mg, 2.0 mmol, 1.2 eq) and NH_4_OH (28%, 1.25 mL) was added dropwise to a solution of alkyne (1.0 eq) in ethanol (5 mL) under a nitrogen atmosphere. The reaction mixture was stirred at room temperature for 15 min. Thereafter, the precipitate was collected via vacuum filtration and washed thoroughly with water. There were no NMR (^1^H or ^13^C) or mass spectra recorded for the cuprous acetylides due to their poor solubility in organic solvents.

#### 3.2.4. General Protocol for Castro–Stephens Coupling via Microwave-Assistance (A)

A mixture of aloe-emodin iodide (1 eq.), cuprous acetylide (2.5–3.0 eq.) in DMF (5 mL) was added to a 10 mL glass vial, flushed with nitrogen, and sealed. The mixture was microwave-irradiated while stirring at 150 °C at a maximum power of 150 W for 30 min. Conversion was monitored via TLC. The solvent was removed under reduced pressure once the mixture was cooled to room temperature. The solid obtained was further purified via column chromatography on silica gel (petroleum ether:EtOAc 7:3) to give the desired anthra[1,2-*b*]furan.

#### 3.2.5. General Protocol for Castro–Stephens Coupling via Microwave-Assistance (B)

A mixture of aloe-emodin iodide (1 eq.), cuprous acetylide (2.5–3.0 eq.) in DMF (5 mL) was added to a 10 mL glass vial, flushed with nitrogen, and sealed. The mixture was microwave-irradiated while stirring at 150 °C at a maximum power of 150 W for 30 min. Conversion was monitored via TLC. Once the mixture was cooled to room temperature, the solvent was removed under reduced pressure to obtain a solid residue, which was further used in Section 3.2.6.

#### 3.2.6. General Protocol for Deprotection

A solution of methanol (10 mL) and HCl (37%, 0.5 mL) was added to the solid residue obtained from Section 3.2.5. The mixture was refluxed overnight. Full conversion was monitored by TLC. Thereafter, the mixture was cooled to room temperature, and the precipitate collected via vacuum filtration, washed with water, and dried in a vacuum oven.

### 3.3. Structural Characterization

#### 3.3.1. (5-Acetoxy-4-hydroxy-9,10-dioxo-9,10-dihydroanthracen-2-yl)methyl Acetate (**2**)

Acetylated aloe-emodin 2 was prepared according to Section 3.2.1. Crystallization of the yellow solid using chloroform–petroleum ether–toluene 1:1:1 mixture afforded a black solid that was 80% compound 2. Further crystallization with chloroform–toluene 1:1.5 led to pure compound **2** as a dark green solid (0.77 g, 2.2 mmol, 58%). R_f_ = 0.90 (DCM/MeOH 9:1). ^1^H NMR (400 MHz, CDCl_3_) δ (ppm): 12.55 (s, 1H), 8.26 (dd, *J* = 7.8, 1.3 Hz, 1H), 7.81 (t, *J* = 7.9 Hz, 1H), 7.73 (d, *J* = 1.8 Hz, 1H), 7.42 (dd, *J* = 8.0, 1.3 Hz, 1H, H7), 7.25 (dd, *J* = 1.7, 0.9 Hz, 1H), 5.17 (s, 2H), 2.47 (s, 3H), 2.18 (s, 3H). ^13^C NMR (101 MHz, CDCl_3_): δ (ppm): 187.54, 181.54, 170.48, 169.55, 162.88, 150.57, 145.73, 135.59, 135.27, 132.92, 130.35, 126.08, 124.62, 122.64, 117.83, 115.98, 64.70, 21.20, 20.85. HRMS ESI: *m*/*z* [M + H]^+^ calculated for C_19_H_14_O_7_: 355.0812; found: 355.0807.


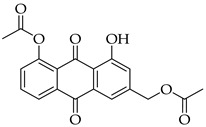


#### 3.3.2. (5-Acetoxy-4-hydroxy-3-iodo-9,10-dioxo-9,10-dihydroanthracen-2-yl)methyl Acetate (**3**)

Aloe-emodin iodide 3 was prepared according to Section 3.2.2. using compound 2 (0.5 g, 1.4 mmol). Crystallization from toluene afforded pure compound **3** as a yellow solid (0.5 g, 1.1 mol, 78%). R_f_ = 0.40 (PE:EtOAc 7:3). Mp = 168–171 °C. ^1^H NMR (400 MHz, CDCl_3_) δ (ppm): 13.64 (s, 1H), 8.30 (dd, *J* = 7.8, 1.3 Hz, 1H), 7.87 (t, *J* = 7.9 Hz, 1H), 7.86–7.78 (m, 1H), 7.47 (dd, *J* = 8.1, 1.3 Hz, 1H), 5.24 (d, *J* = 0.7 Hz, 2H), 2.49 (s, 3H), 2.27 (s, 3H). ^13^C NMR (101 MHz, CDCl_3_) δ (ppm): 187.34, 181.29, 170.30, 169.46, 161.24, 150.74, 148.47, 136.00, 135.12, 132.11, 130.53, 126.07, 124.17, 117.97, 114.94, 98.02, 69.98, 21.17, 20.89. HRMS ESI: *m*/*z* [M + H]^+^ calculated for C_19_H_13_IO_7_: 480.9781; found: 480.9791.


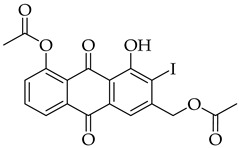


#### 3.3.3. (Phenylethynyl) Copper (**4a**)

Cuprous acetylide **4a** was prepared according to Section 3.2.3. using phenylacetylene (0.18 mL, 1.6 mmol, 1.0 eq). The yellow solid obtained was used without further purification.


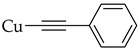


#### 3.3.4. Oct-1-yn-1-yl Copper (**4b**)

Cuprous acetylide **4b** was prepared according to Section 3.2.3. using *n*-octyne (0.25 mL, 1.7 mmol, 1.0 eq). The yellow solid obtained was used without further purification.


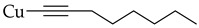


#### 3.3.5. (3-Methoxy-3-oxoprop-1-yn-1-yl)copper (**4c**)

A solution of methyl propiolate (0.1 mL, 1.1 mmol, 1.0 eq) in ethanol (6 mL) was added to a mixture containing CuSO_4_.5H_2_O (300 mg, 1.2 mmol), NH_2_OH.HCl (200 mg, 2.9 mmol), NH_4_OH (28%, 1.20 mL), and water (5 mL) under nitrogen atmosphere. A yellow precipitate formed which was vacuum-filtered and washed thoroughly with water. The yellow solid was used without further purification.


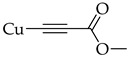


#### 3.3.6. (10-Acetoxy-6,11-dioxo-2-phenyl-6,11-dihydroanthra[1,2-b]furan-4-yl)methyl Acetate (**5a**)

Compound **5a** was prepared according to the Section 3.2.4. using aloe-emodin iodide **3** (50 mg, 0.10 mmol) and (phenylethynyl) copper **5a** (51 mg, 0.31 mmol). Column purification followed by recrystallization from toluene produced a yellow solid (22 mg, 0.05 mmol, 47%). R_f_ = 0.36 (Petroleum Ether: EtOAc 7:3). Mp = 192–196 °C. ^1^H NMR (400 MHz, CDCl_3_) δ (ppm): 8.27 (dd, *J* = 7.8, 1.3 Hz, 1H), 8.16 (s, 1H), 8.04–7.97 (m, 2H), 7.78 (t, *J* = 7.9 Hz, 1H), 7.56–7.49 (m, 2H), 7.49–7.45 (m, 1H), 7.43 (dd, *J* = 8.0, 1.3 Hz, 1H), 7.16 (s, 1H), 5.44 (s, 2H), 2.60 (s, 3H), 2.19 (s, 3H). ^13^C NMR (101 MHz, CDCl3) δ (ppm): 182.12, 180.59, 170.67, 169.82, 161.93, 151.88, 150.10, 135.48, 135.07, 134.64, 134.09, 130.15, 129.99, 129.01, 128.97, 125.93, 125.74, 121.39, 118.96, 99.38, 63.40, 21.39, 20.92. HRMS ESI: *m*/*z* [M + Na]^+^ calculated for C_27_H_18_O_7_: 447.0945; found: 447.0953.


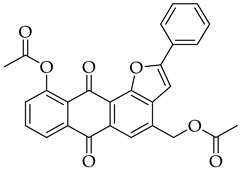


#### 3.3.7. (10-Acetoxy-2-hexyl-6,11-dioxo-6,11-dihydroanthra[1,2-b]furan-4-yl)methyl Acetate (**5b**)

Compound 5b was prepared according to the Section 3.2.4. using aloe-emodin iodide 3 (152 mg, 0.32 mmol) and oct-1-yn-1-ylcopper **4b** (138 mg, 0.84 mmol). Column purification followed by recrystallization from EtOAc produced a yellow solid (50 mg, 0.11 mmol, 34%). R_f_ = 0.66 (Petroleum Ether: EtOAc 7:3). Mp = 128–131 °C. ^1^H NMR (400 MHz, CDCl_3_) δ (ppm): 8.27 (dd, *J* = 7.8, 1.3 Hz, 1H), 8.16 (s, 1H), 7.77 (t, *J* = 7.9 Hz, 1H), 7.41 (dd, *J* = 8.1, 1.3 Hz, 1H), 6.59 (t, *J* = 1.0 Hz, 1H), 5.42–5.36 (m, 2H), 2.99–2.91 (m, 2H), 2.55 (s, 3H), 2.17 (s, 3H), 1.83 (p, *J* = 7.6 Hz, 2H), 1.51–1.42 (m, 2H), 1.36 (tq, *J* = 6.4, 2.8 Hz, 4H), 0.97–0.88 (m, 3H). ^13^C NMR (101 MHz, CDCl3) δ (ppm): 182.36, 180.93, 170.67, 169.78, 167.00, 150.07, 135.42, 135.09, 134.60, 133.59, 129.96, 128.43, 125.71, 125.18, 120.96, 118.65, 100.55, 63.43, 31.51, 28.94, 28.80, 27.32, 22.57, 21.35, 20.89, 14.08. HRMS ESI: *m*/*z* [M + H]^+^ calculated for C_27_H_26_O_7_: 463.1751; found: 463.1758.


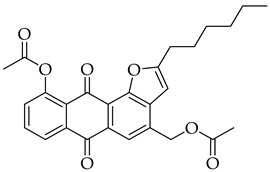


#### 3.3.8. (2-Hexyl-10-hydroxy-6,11-dioxo-6,11-dihydroanthra[1,2-b]furan-4-yl)methyl Acetate (**5c**)

Compound **5c** was prepared according to the Section 3.2.4. using aloe-emodin iodide **3** (152 mg, 0.32 mmol) and oct-1-yn-1-ylcopper **4b** (138 mg, 0.84 mmol). Column purification followed by recrystallization from EtOAc produced a yellow solid (17 mg, 0.10 mmol, 13%). R_f_ = 0.75 (Petroleum Ether: EtOAc 7:3). Mp = 80–83 °C. ^1^H NMR (400 MHz, CDCl_3_) δ (ppm): 12.68 (s, 1H), 8.19 (s, 1H), 7.83 (dd, *J* = 7.5, 1.2 Hz, 1H), 7.66 (t, *J* = 7.9 Hz, 1H), 7.30 (dd, *J* = 8.4, 1.2 Hz, 1H), 6.62 (s, 1H), 5.40 (s, 2H), 3.00–2.92 (m, 2H), 2.18 (s, 3H), 1.85 (p, *J* = 7.6 Hz, 2H), 1.53–1.42 (m, 2H), 1.36 (tt, *J* = 6.0, 2.8 Hz, 4H), 0.91 (td, *J* = 6.0, 5.0, 3.7 Hz, 3H). ^13^C NMR (101 MHz, CDCl3) δ (ppm): 188.19, 182.27, 170.64, 166.90, 162.43, 151.83, 136.58, 135.22, 134.50, 133.29, 129.38, 124.44, 121.44, 119.49, 117.53, 116.40, 100.64, 63.40, 31.49, 28.93, 28.85, 27.46, 22.55, 20.88, 14.06. HRMS ESI: *m*/*z* [M-H]^−^ calculated for C_25_H_24_O_6_: 419.1500; found: 419.1484.


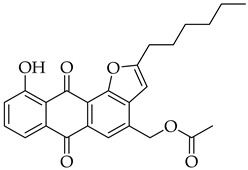


#### 3.3.9. Methyl 10-Acetoxy-4-(acetoxymethyl)-6,11-dioxo-6,11-dihydroanthra[1,2-b]furan-2-carboxylate (**5d**)

Compound **5d** was prepared according to the Section 3.2.4. using aloe-emodin iodide **3** (153 mg, 0.32 mmol) and (3-methoxy-3-oxoprop-1-yn-1-yl)copper **4c** (130 mg, 0.89 mmol). Column purification followed by recrystallization from EtOAc produced a yellow solid (37 mg, 0.08 mmol, 27%). R_f_ = 0.14 (Petroleum Ether: EtOAc 7:3). Mp = 215–218 °C. ^1^H NMR (400 MHz, CDCl_3_) δ (ppm): 8.27 (dd, *J* = 7.8, 1.3 Hz, 1H), 8.24 (d, *J* = 0.9 Hz, 1H), 7.81 (t, *J* = 7.9 Hz, 1H), 7.71 (s, 1H), 7.45 (dd, *J* = 8.0, 1.3 Hz, 1H), 5.45 (d, *J* = 0.8 Hz, 2H), 4.05 (s, 3H), 2.56 (s, 3H), 2.18 (s, 3H). ^13^C NMR (101 MHz, CDCl_3_): δ (ppm): 181.95, 179.86, 170.46, 169.73, 158.95, 152.54, 150.41, 150.28, 136.83, 134.94, 134.64, 132.59, 131.87, 130.54, 125.85, 124.91, 121.55, 120.02, 111.56, 63.15, 52.91, 21.33, 20.84. HRMS ESI: *m*/*z* [M + Na]^+^ calculated for C_23_H_16_O_9_: 459.0687; found: 459.0680.


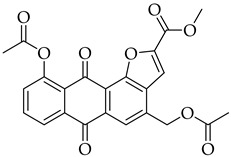


#### 3.3.10. (4-Acetoxy-5-hydroxy-9,10-dioxo-9,10-dihydroanthracen-2-yl)methyl Acetate (**6**)

Acetylated aloe-emodin **6** was prepared according to Section 3.2.1. The chloroform–petroleum ether–toluene 1:1:1 mixture obtained from the crystallization of acetylated aloe-emodin **2** was evaporated to yield a brown solid. Repeated crystallization with chloroform–petroleum ether 1:1 followed by toluene recrystallization yielded pure compound **6** as a yellow solid. R_f_ = 0.90 (DCM/MeOH 9:1). ^1^H NMR (400 MHz, CDCl_3_) δ (ppm): 12.56 (s, 1H), 8.23 (dd, *J* = 1.7, 0.9 Hz, 1H), 7.82 (dd, *J* = 7.5, 1.2 Hz, 1H), 7.68 (dd, *J* = 8.4, 7.5 Hz, 1H), 7.41 (dd, *J* = 1.8, 0.9 Hz, 1H), 7.32 (dd, *J* = 8.4, 1.2 Hz, 1H), 5.26 (s, 1H), 2.50 (s, 3H), 2.21 (s, 3H). ^13^C NMR (101 MHz, CDCl_3_) δ (ppm): 187.53, 181.51, 170.43, 169.51, 162.69, 150.84, 144.65, 136.70, 135.51, 132.64, 128.57, 124.99, 124.43, 124.04, 116.52, 64.34, 21.19, 20.84. HRMS ESI: *m*/*z* [M + H]^−^ calculated for C_19_H_14_O_7_: 353.0667; found: 353.0637.


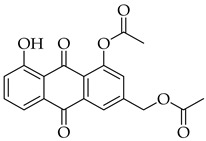


#### 3.3.11. (4-Acetoxy-5-hydroxy-6-iodo-9,10-dioxo-9,10-dihydroanthracen-2-yl)methyl Acetate (**7**)

Aloe-emodin iodide **7** was prepared using the Section 3.2.2. using compound **6** (0.5 g, 1.4 mmol). Crystallization from toluene afforded pure compound **7** as an orange solid (0.5 g, 1.1 mol, 77%). R_f_ = 0.40 (PE:EtOAc 7:3). Mp = 200–205 °C. ^1^H NMR (400 MHz, CDCl_3_) δ (ppm): 13.48 (s, 1H), 8.24–8.21 (m, 1H), 8.20 (s, 1H), 7.56 (d, *J* = 8.0 Hz, 1H), 7.44 (dd, *J* = 1.8, 0.9 Hz, 1H), 5.26 (t, *J* = 0.7 Hz, 2H), 2.49 (s, 3H), 2.21 (s, 3H). ^13^C NMR (101 MHz, CDCl_3_) δ (ppm): 187.26, 181.13, 170.40, 169.40, 161.28, 151.02, 146.35, 145.19, 135.32, 132.58, 128.76, 124.42, 123.48, 120.41, 115.80, 95.72, 64.29, 21.16, 20.84. HRMS ESI: *m*/*z* [M] calculated for C_19_H_13_IO_7_: 479.9708; found: 479.9725.


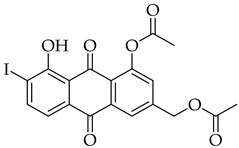


#### 3.3.12. (10-Acetoxy-6,11-dioxo-2-phenyl-6,11-dihydroanthra[1,2-b]furan-8-yl)methyl Acetate (**8a**)

Compound **8a** was prepared according to the Section 3.2.4. using aloe-emodin iodide **7** (158 mg, 0.33 mmol) and (phenylethynyl) copper **4a** (159 mg, 0.97 mmol). Column purification followed by recrystallization from toluene produced a yellow solid (53 mg, 0.12 mmol, 36%). R_f_ = 0.29 (Petroleum Ether: EtOAc 7:3). Mp = 180–184 °C. ^1^H NMR (400 MHz, CDCl_3_) δ (ppm): 8.23 (dt, *J* = 1.7, 0.7 Hz, 1H), 8.19 (d, *J* = 8.1 Hz, 1H), 8.04–7.99 (m, 2H), 7.90 (d, *J* = 8.1 Hz, 1H), 7.52 (dd, *J* = 8.3, 6.8 Hz, 2H), 7.48–7.43 (m, 1H), 7.40 (dd, *J* = 1.7, 0.8 Hz, 1H), 7.11 (s, 1H), 5.24 (s, 2H), 2.60 (s, 3H), 2.19 (s, 3H). ^13^C NMR (101 MHz, CDCl3): δ (ppm): 182.13, 180.61, 170.52, 169.77, 161.78, 151.80, 150.40, 143.49, 137.15, 135.36, 129.99, 129.00, 128.38, 126.22, 125.85, 124.56, 124.28, 122.41, 119.27, 101.05, 64.54, 21.39, 20.88. HRMS ESI: *m*/*z* [M + Na]^+^ calculated for C_27_H_18_O_7_: 447.0945; found: 447.0949.


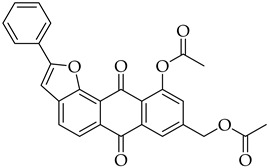


#### 3.3.13. (10-Acetoxy-2-hexyl-6,11-dioxo-6,11-dihydroanthra[1,2-b]furan-8-yl)methyl Acetate (8b)

Compound **8b** was prepared according to the Section 3.2.4. using aloe-emodin iodide **7** (152 mg, 0.32 mmol) and oct-1-yn-1-ylcopper **4b** (138 mg, 0.84 mmol). Column purification followed by recrystallization from EtOAc produced a yellow solid (33 mg, 0.07 mmol, 22%). R_f_ = 0.68 (Petroleum Ether: EtOAc 7:3). Mp = 124–125 °C. ^1^H NMR (400 MHz, CDCl_3_) δ (ppm): 8.23 (d, *J* = 1.8 Hz, 1H), 8.16 (d, *J* = 8.1 Hz, 1H), 7.82 (d, *J* = 8.1 Hz, 1H), 7.38 (d, *J* = 1.9 Hz, 1H), 6.52 (d, *J* = 1.1 Hz, 1H), 5.23 (s, 2H), 3.00–2.89 (m, 2H), 2.56 (s, 3H), 2.18 (s, 3H), 1.83 (p, *J* = 7.6 Hz, 2H), 1.45 (q, *J* = 7.0 Hz, 2H), 1.36 (tt, *J* = 7.4, 3.5 Hz, 4H), 0.98–0.84 (m, 3H). ^13^C NMR (101 MHz, CDCl3): δ (ppm): 182.33, 169.74, 166.73, 151.57, 150.36, 143.41, 137.18, 135.38, 128.35, 125.74, 124.24, 121.99, 102.21, 64.53, 31.52, 28.92, 28.74, 27.31, 22.57, 21.34, 20.87, 14.08. HRMS ESI: *m*/*z* [M + H]^+^ calculated for C_27_H_26_O_7_: 463.1751; found: 463.1759.


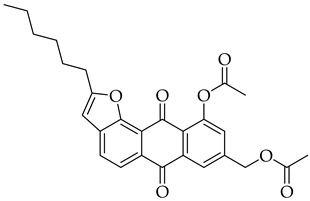


#### 3.3.14. (2-Hexyl-10-hydroxy-6,11-dioxo-6,11-dihydroanthra[1,2-b]furan-8-yl)methyl Acetate (**8c**)

Compound **8c** was prepared according to the Section 3.2.4. using aloe-emodin iodide **7** (152 mg, 0.32 mmol) and oct-1-yn-1-ylcopper **4b** (138 mg, 0.84 mmol). Column purification followed by recrystallization from EtOAc produced a yellow solid (13 mg, 0.03 mmol, 10%). R_f_ = 0.79 (Petroleum Ether: EtOAc 7:3). Mp = 119–121 °C. ^1^H NMR (400 MHz, CDCl_3_) δ (ppm): 12.69 (s, 1H), 8.17 (d, *J* = 8.1 Hz, 1H), 7.84 (d, *J* = 8.1 Hz, 1H), 7.76 (d, *J* = 1.7 Hz, 1H), 7.24 (dd, *J* = 1.7, 0.9 Hz, 1H), 6.54 (s, 1H), 5.18 (s, 2H), 2.98–2.89 (m, 2H), 2.18 (s, 3H), 1.83 (p, *J* = 7.6 Hz, 2H), 1.52–1.41 (m, 2H), 1.35 (pd, *J* = 5.1, 4.0, 2.2 Hz, 4H), 0.90 (td, *J* = 5.9, 4.9, 2.7 Hz, 3H). ^13^C NMR (101 MHz, CDCl3): δ (ppm): 188.16, 182.22, 170.53, 166.64, 162.62, 151.69, 145.48, 136.99, 133.63, 129.22, 126.47, 122.50, 122.14, 117.89, 117.74, 115.85, 102.26, 64.88, 31.51, 28.92, 28.78, 27.44, 22.56, 20.87, 14.07. HRMS ESI: *m*/*z* [M + H]^+^ calculated for C_25_H_24_O_6_: 421.1646; found: 421.1650.


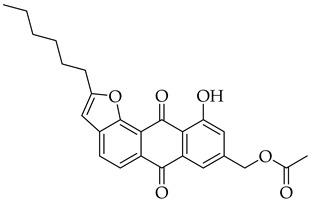


#### 3.3.15. 1,8-Dihydroxy-3-(hydroxymethyl)-2-iodoanthracene-9,10-dione (**9**)

Compound 9 was prepared according to the Section 3.2.6. using aloe-emodin iodide 3 (107 mg, 0.22 mmol) to yield an orange solid (85 mg, 0.21 mmol, 96%). R_f_ = 0.4 (PE:EtOAc 7:3). Mp = 241–243 °C. ^1^H NMR (400 MHz, DMSO-*d_6_*) δ (ppm) 12.82 (s, 1H), 11.71 (s, 1H), 7.82–7.73 (m, 2H), 7.66 (dd, J = 7.5, 1.2 Hz, 1H), 7.35 (dd, J = 8.4, 1.2 Hz, 1H), 5.85 (t, J = 5.5 Hz, 1H), 4.47 (d, J = 5.0 Hz, 2H). ^13^C NMR (101 MHz, DMSO-*d_6_)* δ (ppm) 191.84, 181.70, 161.77, 160.08, 155.30, 138.07, 133.68, 132.64, 125.02, 119.88, 117.71, 116.08, 114.34, 97.27, 68.59. HRMS ESI: *m*/*z* [M + 1]^+^ calculated for C_15_H_9_IO_5_: 396.9569; found: 396.9583.


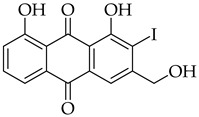


#### 3.3.16. 10-Hydroxy-4-(hydroxymethyl)-2-phenylanthra[1,2-b]furan-6,11-dione (**10a**)

Compound **10a** was prepared according to the Section 3.2.5. using aloe-emodin iodide **3** (152 mg, 0.32 mmol) and (phenylethynyl) copper **4a** (166 mg, 1.01 mmol). Recrystallization from toluene produced an orange solid (81 mg, 0.22 mmol, 69%). It had very poor solubility in acetone, acetonitrile, chloroform, DMSO, methanol, toluene, and water. R_f_ = 0.57 (DCM:MeOH 92:8). Mp = 291–292 °C. ^1^H NMR (600 MHz, DMSO-*d_6_*) δ (ppm) 12.61 (s, 1H), 8.15 (s, 1H), 8.06 (d, *J* = 7.4 Hz, 2H), 7.79 (d, *J* = 7.7 Hz, 1H), 7.77 (s, 1H), 7.72 (dd, *J* = 7.5, 1.3 Hz, 1H), 7.59 (t, *J* = 7.7 Hz, 2H), 7.51 (t, *J* = 7.4 Hz, 1H), 7.38 (dd, *J* = 8.2, 1.2 Hz, 1H), 5.75–5.70 (m, 1H), 4.92 (d, *J* = 5.9 Hz, 2H). HRMS ESI: *m*/*z* [M + 1]^+^ calculated for C_23_H_14_O_5_: 371.0914; found: 371.0911.


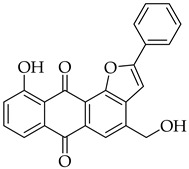


#### 3.3.17. 2-Hexyl-10-hydroxy-4-(hydroxymethyl)anthra[1,2-b]furan-6,11-dione (**10b**)

Compound **10b** was prepared according to the Section 3.2.5. using aloe-emodin iodide **3** (152 mg, 0.32 mmol) and oct-1-yn-1-ylcopper **4b** (138 mg, 0.84 mmol) to yield a yellow solid (57 mg, 0.15 mmol, 48%). **R_f_** = 0.36 (PE:EtOAc 7:3). **Mp** = 143–145 °C. It had poor solubility in chloroform and water. **^1^H NMR** (400 MHz, CDCl_3_) δ (ppm) 12.65 (s, 1H), 8.05 (s, 1H), 7.75 (dd, *J* = 7.5, 1.2 Hz, 1H), 7.63 (dd, *J* = 8.4, 7.5 Hz, 1H), 7.27 (dd, *J* = 8.4, 1.2 Hz, 1H), 6.61 (d, *J* = 1.0 Hz, 1H), 4.96 (d, *J* = 5.6 Hz, 2H), 2.94–2.86 (m, 2H), 2.33 (t, *J* = 6.1 Hz, 1H), 1.81 (p, *J* = 7.6 Hz, 2H), 1.52–1.42 (m, 2H), 1.35 (tt, *J* = 6.5, 3.0 Hz, 4H), 0.96–0.86 (m, 3H). **^13^C NMR** (101 MHz, CDCl_3_) δ (ppm): 188.04, 182.39, 166.53, 162.31, 151.74, 139.59, 136.43, 134.72, 133.19, 129.19, 124.36, 120.00, 119.38, 116.96, 116.26, 100.77, 63.20, 31.52, 28.97, 28.78, 27.36, 22.56, 14.08. **HRMS** ESI: *m*/*z* [M + 1]^+^ calculated for C_23_H_22_O_5_: 379.1540; found: 379.1551.


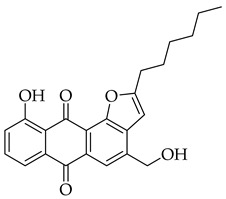


#### 3.3.18. Methyl 10-hydroxy-4-(hydroxymethyl)-6,11-dioxo-6,11-dihydroanthra[1,2-b]furan-2-carboxylate (**10c**)

Compound **10c** was prepared according to the Section 3.2.5. using aloe-emodin iodide **3** (151 mg, 0.31 mmol) and (3-methoxy-3-oxoprop-1-yn-1-yl)copper **4c** (129 mg, 0.88 mmol). The solid obtained was purified by column chromatography on silica gel (DCM 100% → DCM:MeOH 98:2) to give a yellow solid (59 mg, 0.17 mmol, 54%). It had poor solubility in acetone and water. R_f_ = 0.12 (PE:EtOAc 7:3). Mp = 269–271 °C. ^1^H NMR (600 MHz, 60 °C, DMSO-*d_6_*) δ (ppm) 12.37 (s, 1H), 8.19 (d, *J* = 1.0 Hz, 1H), 8.02 (s, 1H), 7.80 (dd, *J* = 8.3, 7.5 Hz, 1H), 7.73 (dd, *J* = 7.5, 1.2 Hz, 1H), 7.38 (dd, *J* = 8.3, 1.2 Hz, 1H), 5.65 (t, *J* = 5.7 Hz, 1H), 4.98–4.94 (m, 2H), 3.99 (s, 3H). ^13^C NMR (151 MHz, 60 °C, DMSO-*d_6_*) δ (ppm) 187.07, 182.25, 161.80, 159.00, 152.37, 149.50, 146.11, 137.30, 133.31, 132.90, 131.65, 124.78, 119.59, 119.49, 118.05, 116.64, 112.83, 61.28, 53.07. HRMS ESI: *m*/*z* [M + 1]^+^ calculated for C_19_H_12_O_7_: 353.0656; found: 353.0660.


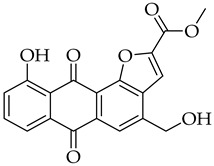


#### 3.3.19. 1,8-Dihydroxy-6-(hydroxymethyl)-2-iodoanthracene-9,10-dione (**11**)

Compound **11** was prepared according to the Section 3.2.6. using aloe-emodin iodide **7** (104 mg, 0.22 mmol) to yield an orange solid (77 mg, 0.20 mmol, 90%). **R_f_** = 0.30 (PE:EtOAc 7:3). Mp = 261–263 °C. ^1^H NMR (400 MHz, DMSO-*d_6_*) δ (ppm) 12.84 (s, 1H), 11.69 (s, 1H), 8.29 (d, *J* = 8.0 Hz, 1H), 7.64 (d, *J* = 1.6 Hz, 1H), 7.40 (d, *J* = 8.0 Hz, 1H), 7.28 (d, *J* = 1.4 Hz, 1H), 4.62 (s, 2H). ^13^C NMR (101 MHz, DMSO-*d_6_*) δ (ppm) 191.60, 181.55, 162.08, 160.43, 154.50, 146.73, 133.52, 133.42, 121.26, 120.73, 117.62, 115.80, 114.55, 96.51, 62.51. HRMS ESI: *m*/*z* [M + 1]^+^ calculated for C_15_H_9_IO_5_: 396.9569; found: 396.9577.


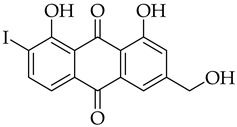


#### 3.3.20. 10-Hydroxy-8-(hydroxymethyl)-2-phenylanthra[1,2-b]furan-6,11-dione (**12a**)

Compound **12a** was prepared according to the Section 3.2.5. using aloe-emodin iodide **7** (150 mg, 0.31 mmol) and (phenylethynyl) copper **4a** (160 mg, 0.97 mmol). Recrystallization from toluene yielded a red solid (76 mg, 0.21 mmol, 66%). Poor solubility in acetone, acetonitrile, chloroform, methanol and water. R_f_ = 0.31 (PE:EtOAc 7:3). Mp = 280–284 °C. ^1^H NMR (400 MHz, DMSO-*d_6_*) δ (ppm) 12.51 (s, 1H), 8.00 (t, *J* = 7.2 Hz, 4H), 7.62–7.53 (m, 4H), 7.50 (t, *J* = 7.3 Hz, 1H), 7.23–7.18 (m, 1H), 5.57 (t, *J* = 5.8 Hz, 1H), 4.59 (d, *J* = 5.7 Hz, 2H). ^13^C NMR (101 MHz, DMSO-*d_6_*) δ (ppm) 187.47, 181.99, 162.07, 160.88, 153.36, 151.40, 136.91, 133.11, 130.54, 129.84, 129.65, 129.04, 127.73, 125.80, 122.66, 120.78, 118.15, 117.06, 115.15, 102.38, 62.61. HRMS ESI: *m*/*z* [M + 1]^+^ calculated for C_23_H_14_O_5_: 371.0914; found: 371.0920.


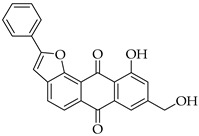


#### 3.3.21. 2-Hexyl-10-hydroxy-8-(hydroxymethyl)anthra[1,2-b]furan-6,11-dione (**12b**)

Compound **12b** was prepared according to the Section 3.2.5. using aloe-emodin iodide **7** (152 mg, 0.32 mmol) and oct-1-yn-1-ylcopper **4b** (138 mg, 0.84 mmol). Recrystallization from toluene yielded a brown solid (57 mg, 0.15 mmol, 48%). R_f_ = 0.36 (PE:EtOAc 7:3). Mp = 187–190 °C. ^1^H NMR (600 MHz, 40 °C, CDCl_3_) δ (ppm) 12.69 (s, 1H), 8.19 (d, *J* = 8.1 Hz, 1H), 7.85 (d, *J* = 8.1 Hz, 1H), 7.79 (d, *J* = 1.7 Hz, 1H), 7.33 (dd, *J* = 1.7, 0.9 Hz, 1H), 6.56 (d, *J* = 1.1 Hz, 1H), 4.82 (d, *J* = 5.7 Hz, 2H), 3.00–2.94 (m, 2H), 2.00 (t, *J* = 6.0 Hz, 1H), 1.87 (p, *J* = 7.6 Hz, 2H), 1.53–1.46 (m, 1H), 1.44–1.33 (m, 4H), 0.97–0.91 (m, 3H). ^13^C NMR (151 MHz, 40 °C, CDCl_3_) δ (ppm) 188.10, 182.40, 166.56, 162.86, 151.72, 150.60, 136.92, 133.56, 129.36, 126.25, 122.43, 121.17, 117.91, 117.15, 115.56, 102.19, 64.29, 31.47, 28.88, 28.76, 27.44, 22.50, 13.97. HRMS ESI: *m*/*z* [M + 1]^+^ calculated for C_23_H_22_O_5_: 379.1540; found: 379.1546.


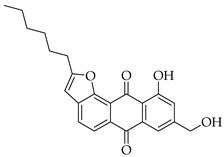


#### 3.3.22. Methyl 10-Hydroxy-8-(hydroxymethyl)-6,11-dioxo-6,11-dihydroanthra[1,2-b]furan-2-carboxylate (**12c**)

Compound **12c** was prepared according to the Section 3.2.5. using aloe-emodin iodide **7** (303 mg, 0.63 mmol) and (3-methoxy-3-oxoprop-1-yn-1-yl)copper **4c** (265 mg, 0.88 mmol). Recrystallization from toluene yielded a brown solid (120 mg, 0.34 mmol, 54%). It had poor solubility in acetone, acetonitrile, chloroform, methanol, and water. **R_f_** = 0.92 (DCM:MeOH 92:8). Mp = 286–288 °C. ^1^H NMR (400 MHz, DMSO-*d_6_*) δ (ppm) 12.30 (s, 1H), 8.22 (d, *J* = 8.2 Hz, 1H), 8.09 (d, *J* = 8.2 Hz, 1H), 7.89 (s, 1H), 7.62 (d, *J* = 1.6 Hz, 1H), 7.24 (d, *J* = 1.5 Hz, 1H), 5.57 (t, *J* = 5.8 Hz, 1H), 4.60 (d, *J* = 5.6 Hz, 2H), 3.94 (s, 3H). ^13^C NMR (101 MHz, DMSO-*d_6_*) δ (ppm)186.72, 182.11, 161.98, 158.95, 153.55, 152.05, 149.75, 134.24, 132.89, 132.71, 130.29, 122.84, 121.09, 119.34, 117.21, 115.08, 114.18, 62.56, 53.20. HRMS ESI: *m*/*z* [M + 1]^+^ calculated for C_19_H_12_O_7_: 353.0656; found: 353.0657.


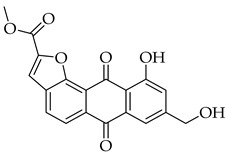


### 3.4. Cytotoxic Activity by CCK8 Assay

The stock solutions of all the aloe-emodin derivatives with specific concentrations were prepared in DMSO and diluted to working concentrations with phosphate-buffered saline (PBS) when conducting the cytotoxicity experiment. The final ratio of DMSO was strictly controlled within a safe range, typically below 0.1%. All the measured IC_50_ values fall within the above concentration ranges, ensuring the reproducibility and reliability of the data. The concentration ranges used for the cytotoxicity determination were as follows:
A549, HepG2, Skov3, and MCF-7 cells:2.5, 5, 10, 20, 40, and 80 µMHCT116 cells:100, 200, 300, 400, 500, and 600 µM

Cells were seeded into 96-well plates at a density of 5 × 10^3^ cells per well in complete culture medium. After cell adherence, different concentrations of the drug were added to each well. Following 3 h of incubation, the respective treatments were applied to each group. After an additional 24 h incubation, 10 μL of CCK8 (Cell Counting Kit-8,Biosharp Life Science, Hefei Anhui, China) solution was added to each well and the cells were further cultured for 2 h. The absorbance at 450 nm was measured using a microplate reader (Biotek, TEK, MQX200, BioTek Instruments, Inc., Winooski, VT, USA) to evaluate cell viability.

## 4. Conclusions

In this study, several anthra[1,2-*b*]furans were synthesized starting from the parent compound aloe-emodin. Preliminary analyses were performed on these derivatives by evaluating their cytotoxic activity against five cancer cell lines. Preliminary analysis showed that anthra[1,2-*b*]furan **10c** exhibited improved cytotoxic activity against all cancer cell lines compared with all the other derivatives and the parent compound, aloe-emodin **1**. Anthra[1,2-*b*]furan **10c** could be a promising hit meriting future consideration for further biological studies. Future cytotoxic analysis against normal cells would give insight into the selectivity of **10c** for cancer cells instead of healthy cells. Furthermore, mechanism studies against A549 and MCF-7 would elucidate the signaling pathways utilized to target cancer cells. Molecular docking studies of C2- and C7-functionalized derivatives with focus on methoxy carbonyl substituents could give insight into their interaction with DNA or protein structures expressed in cancer cells. We can also look out to expand the current library to gain more insight into the correlation between functional groups and cytotoxic activity. Expansion of the current library to include both acetylated and non-acetylated anthra[1,2-b]furans would give clarity on the importance of 1- and 8-hydroxyl groups. In addition, the scope of the library could be increased with a focus on the use of the methoxy carbonyl as a spacer group to synthesize derivatives with improved cytotoxic activity.

## Figures and Tables

**Table 1:** Optimization reaction for anthra[1,2-*b*]furan **5a**.

Entry	4a (eq.)	Solvent Type	Base	Time (h)	Temperature (°C)	Yield (%)
1	1.5	DMF	-	1.5	Reflux	20
2	1.5	DMF	-	4.0	150	21
3	1.5	DMF	-	48	110	10
4	3.0	DMF	-	1.5	150	30
5	3.0	DMF	-	0.5	MW, 150	47
6	3.0	Toluene	-	72	90	26
7	3.0	1,4-dioxane	-	24	Reflux	20
8	3.0	Toluene	DMEDA	16	90	-

**Table 2:** In vitro cytotoxic activity of aloe-emodin derivatives.

Compound	IC_50_ (μM) ^1^				
	A549 ^2^	HCT116 ^3^	HepG2 ^4^	Skov3 ^5^	MCF-7 ^6^
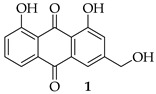	28.25 ± 0.30	217.10 ± 5.46	46.14 ± 1.02	47.71 ± 2.36	36.12 ± 0.72
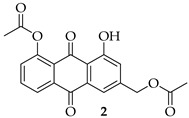	36.93 ± 0.46	223.83 ± 2.01	54.23 ± 1.39	57.03 ± 3.25	48.51 ± 1.27
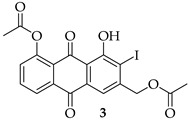	15.45 ± 0.13	209.60 ± 3.03	29.98 ± 1.46	38.05 ± 0.43	21.92 ± 1.65
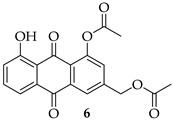	21.24 ± 0.06	213.47 ± 2.50	41.91 ± 0.95	43.78 ± 2.47	29.40 ± 0.67
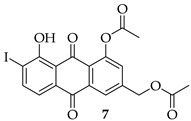	44.30 ± 0.37	221.07 ± 1.96	61.60 ± 1.64	69.56 ± 4.32	49.40 ± 1.98
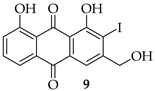	13.26 ± 0.29	194.43 ± 3.69	30.66 ± 0.16	34.25 ± 1.17	20.08 ± 0.30
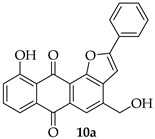	108.37 ± 7.85	286.87 ± 3.54	141.37 ± 19.15	126.63 ± 25.62	99.43 ± 4.83
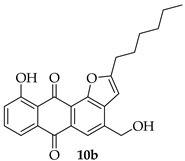	47.20 ± 1.26	261.33 ± 1.40	61.65 ± 2.39	70.67 ± 7.90	53.33 ± 1.07
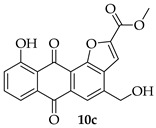	0.49 ± 0.05	175.87 ± 2.50	20.63 ± 0.55	10.85 ± 1.68	2.91 ± 0.15
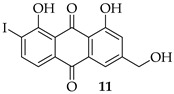	53.26 ± 2.75	279.03 ± 3.78	70.17 ± 3.23	75.22 ± 11.41	56.74 ± 3.51
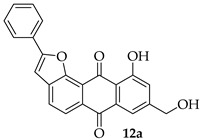	33.38 ± 0.62	220.33 ± 1.54	48.47 ± 3.42	54.29 ± 1.92	41.51 ± 1.13
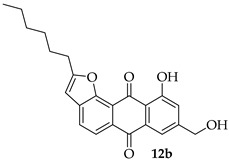	27.94 ± 1.09	216.03 ± 0.35	40.67 ± 1.14	47.65 ± 5.75	32.51 ± 0.50
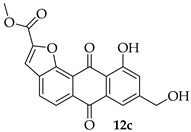	20.99 ± 0.60	212.53 ± 3.61	31.93 ± 2.26	41.56 ± 2.45	25.81 ± 1.82

^1^ IC_50_ is the compound concentration which inhibits 50% of the cancer cell growth, determined by CCK8 method; ^2^ lung adenocarcinoma; ^3^ colorectal carcinoma; ^4^ liver cancer cell; ^5^ ovarian cancer cells; and ^6^ breast cancer cell.

## Data Availability

The original contributions presented in this study are included in the article/Supplementary Materials. Further inquiries can be directed to the corresponding author.

## Supplementary Materials

The following supporting information can be downloaded at: https://www.mdpi.com/article/10.3390/molecules31101676/s1. Figure S1: ^1^H, 400 Hz, CDCl_3_, **2**; Figure S2: ^13^C, 101 MHz, CDCl_3_, **2**; Figure S3: ^1^H, 400 Hz, CDCl_3_, **3**; Figure S4: ^13^C, 101 MHz, CDCl_3_, **3**; Figure S5: ^1^H, 400 Hz, CDCl_3_, **5a**; Figure S6: ^13^C, 101 MHz, CDCl_3_, **5a**; Figure S7: ^1^H, 400 Hz, CDCl_3_, **5b**; Figure S8: ^13^C, 101 MHz, CDCl_3_, **5b**; Figure S9: ^1^H, 400 Hz, CDCl_3_, **5c**; Figure S10: ^13^C, 101 MHz, CDCl_3_, **5c**; Figure S11: ^1^H, 400 Hz, CDCl_3_, **5d**; Figure S12: ^13^C, 101 MHz, CDCl_3_, **5d**; Figure S13: ^1^H, 400 Hz, CDCl_3_, **6**; Figure S14: ^13^C, 101 MHz, CDCl_3_, **6**; Figure S15: ^1^H, 400 Hz, CDCl_3_, **7**; Figure S16: ^13^C, 101 MHz, CDCl_3_, **7**; Figure S17: ^1^H, 400 Hz, CDCl_3_, **8a**; Figure S18: ^13^C, 101 MHz, CDCl_3_, **8a**; Figure S19: ^1^H, 400 Hz, CDCl_3_, **8b**; Figure S20: ^13^C, 101 MHz, CDCl_3_, **8b**; Figure S21: ^1^H, 400 Hz, CDCl_3_, **8c**; Figure S22: ^13^C, 101 MHz, CDCl_3_, **8c**; Figure S23: ^1^H, 400 Hz, DMSO-*d_6_*, **9**; Figure S24: ^13^C, 101 MHz, DMSO-*d_6_*, **9**; Figure S25: ^1^H, 400 Hz, DMSO-*d_6_*, **10a**; Figure S26: ^1^H, 400 Hz, CDCl_3_, **10b**; Figure S27: ^13^C, 101 MHz, CDCl_3_, **10b**; Figure S28: ^1^H, 600 Hz, 60 °C, DMSO-*d_6_*, **10c**; Figure S29: ^13^C, 151 MHz, 60 °C, DMSO-*d_6_*, **10c**; Figure S30: ^1^H, 400 Hz, DMSO-*d_6_*, **11**; Figure S31: ^13^C, 101 MHz, DMSO-*d_6_*, **11**; Figure S32: ^1^H, 400 Hz, DMSO-*d_6_*_3_, **12a**; Figure S33: ^13^C, 101 MHz, DMSO-*d_6_*, **12a**; Figure S34: ^1^H, 600 Hz, 40 °C, CDCl_3_, **12b**; Figure S35: ^13^C, 151 MHz, 40 °C, CDCl_3_, **12b**; Figure S36: ^1^H, 400 Hz, DMSO-*d_6_*, **12c**; Figure S37: ^13^C, 101 MHz, CDCl_3_, **12c**.

## References

[1] Dong X., Zeng Y., Liu Y., You L., Yin X., Fu J., Ni J. (2020). Aloe-Emodin: A Review of Its Pharmacology, Toxicity, and Pharmacokinetics. Phytother. Res..

[2] Sanders B., Ray A.M., Goldberg S., Clark T., McDaniel H.R., Atlas S.E., Farooqi A., Konefal J., Lages L.C., Lopez J. (2017). Anti-Cancer Effects of Aloe-Emodin: A Systematic Review. J. Clin. Transl. Res..

[3] Şeker Karatoprak G., Küpeli Akkol E., Yücel Ç., Bahadir Acikara Ö., Sobarzo-Sánchez E. (2022). Advances in Understanding the Role of Aloe Emodin and Targeted Drug Delivery Systems in Cancer. Oxid. Med. Cell. Longev..

[4] Park M.Y., Kwon H.J., Sung M.K. (2009). Evaluation of Aloin and Aloe-Emodin as Anti-Inflammatory Agents in Aloe by Using Murine Macrophages. Biosci. Biotechnol. Biochem..

[5] Qun T., Zhou T., Hao J., Wang C., Zhang K., Xu J., Wang X., Zhou W. (2023). Antibacterial Activities of Anthraquinones: Structure-Activity Relationships and Action Mechanisms. RSC Med. Chem..

[6] Li Z., Yao R., Guo H., Jing W., Guo X., Liu X., Pan Y., Cao P., Zhang L., Yang J. (2025). Research Progress on Chemical Compositions, Pharmacological Activities, and Toxicities of Quinone Compounds in Traditional Chinese Medicines. Toxics.

[7] Nowak-Perlak M., Bromke M.A., Ziółkowski P., Woźniak M. (2022). The Comparison of the Efficiency of Emodin and Aloe-Emodin in Photodynamic Therapy. Int. J. Mol. Sci..

[8] Spiegel M. (2025). On the Photosensitizing Properties of Aloe-Emodin in Photodynamic Therapy: Insights from the Molecular Modeling. J. Phys. Chem. B.

[9] Lin J.G., Chen G.W., Li T.M., Chouh S.T., Tan T.W., Chung J.G. (2006). Aloe-Emodin Induces Apoptosis in T24 Human Bladder Cancer Cells through the P53 Dependent Apoptotic Pathway. J. Urol..

[10] Huang P.H., Huang C.Y., Chen M.C., Lee Y.T., Yue C.H., Wang H.Y., Lin H. (2013). Emodin and Aloe-Emodin Suppress Breast Cancer Cell Proliferation through ER α Inhibition. Evid.-Based Complement. Altern. Med..

[11] Guo J.M., Xiao B.X., Liu Q., Zhang S., Liu D.H., Gong Z.H. (2007). Anticancer Effect of Aloe-Emodin on Cervical Cancer Cells Involves G 2/M Arrest and Induction of Differentiation. Acta Pharmacol. Sin..

[12] Suboj P., Babykutty S., Srinivas P., Gopala S. (2012). Aloe Emodin Induces G2/M Cell Cycle Arrest and Apoptosis via Activation of Caspase-6 in Human Colon Cancer Cells. Pharmacology.

[13] Chen S.H., Lin K.Y., Chang C.C., Fang C.L., Lin C.P. (2007). Aloe-Emodin-Induced Apoptosis in Human Gastric Carcinoma Cells. Food Chem. Toxicol..

[14] Tabolacci C., Oliverio S., Lentini A., Rossi S., Galbiati A., Montesano C., Mattioli P., Provenzano B., Facchiano F., Beninati S. (2011). Aloe-Emodin as Antiproliferative and Differentiating Agent on Human U937 Monoblastic Leukemia Cells. Life Sci..

[15] Kuo P.-L., Lin T.-C., Lin C.-C. (2002). The Antiproliferative Activity of Aloe-Emodin Is through P53-Dependent and P21-Dependent Apoptotic Pathway in Human Hepatoma Cell Lines. Life Sci..

[16] Lee H.-Z. (2001). Protein Kinase C Involvement in Aloe-Emodin- and Emodin-Induced Apoptosis in Lung Carcinoma Cell. Br. J. Pharmacol..

[17] Lee H.-Z., Hsu S.-L., Liu M.-C., Wu C.-H. (2001). Effects and Mechanisms of Aloe-Emodin on Cell Death in Human Lung Squamous Cell Carcinoma. Eur. J. Pharmacol..

[18] Lin M.L., Lu Y.C., Chung J.G., Li Y.C., Wang S.G., NG S.H., Wu C.Y., Su H.L., Chen S.S. (2010). Aloe-Emodin Induces Apoptosis of Human Nasopharyngeal Carcinoma Cells via Caspase-8-Mediated Activation of the Mitochondrial Death Pathway. Cancer Lett..

[19] Lin M.L., Lu Y.C., Chung J.G., Wang S.G., Lin H.T., Kang S.E., Tang C.H., Ko J.L., Chen S.S. (2010). Down-Regulation of MMP-2 through the P38 MAPK-NF-ΚB-Dependent Pathway by Aloe-Emodin Leads to Inhibition of Nasopharyngeal Carcinoma Cell Invasion. Mol. Carcinog..

[20] Pecere T., Gazzola M.V., Mucignat C., Parolin C., Dalla Vecchia F., Cavaggioni A., Basso G., Diaspro A., Salvato B., Carli M. (2000). Aloe-Emodin Is a New Type of Anticancer Agent with Selective Activity Against Neuroectodermal Tumors. Cancer Res..

[21] Xiao B., Guo J., Liu D., Zhang S. (2007). Aloe-Emodin Induces In Vitro G2/M Arrest and Alkaline Phosphatase Activation in Human Oral Cancer KB Cells. Oral. Oncol..

[22] He T.P., Yan W.H., Mo L.E., Liang N.C. (2008). Inhibitory Effect of Aloe-Emodin on Metastasis Potential in HO-8910PM Cell Line. J. Asian Nat. Prod. Res..

[23] Liu K., Park C., Li S., Lee K.W., Liu H., He L., Soung N.K., Ahn J.S., Bode A.M., Dong Z. (2012). Aloe-Emodin Suppresses Prostate Cancer by Targeting the MTOR Complex 2. Carcinogenesis.

[24] Chiu T.H., Lai W.W., Hsia T.C., Yang J.S., Lai T.Y., Wu P.P., Ma C.Y., Yeh C.C., Ho C.C., Lu H.F. (2009). Aloe-Emodin Induces Cell Death Through S-Phase Arrest and Caspase-Dependent Pathways in Human Tongue Squamous Cancer SCC-4 Cells. Anticancer Res..

[25] Long X., Yang P., Chen L., Zhong W., Chen S., Li Y., Lin S., Tian W. (2023). Novel Aloe Emodin–Hydroxyethyl Piperazine Hybrid Dihydrochloride Induces Oral Cancer CAL-27 Cells Apoptosis Through ROS Production, DNA Damage and Mitochondrial Pathways. Med. Chem. Res..

[26] Zhang Q., Wang J., Lan F., Zhai H., Li F., Ma T., Li D., Hou H. (2023). Synthesis and DNA Interaction of Aloe-Emodin α-Amino Phosphate Derivatives. J. Mol. Struct..

[27] Shi D.H., Huang W., Li C., Wang L.T., Wang S.F. (2013). Synthesis, Biological Evaluation and Molecular Modeling of Aloe-Emodin Derivatives as New Acetylcholinesterase Inhibitors. Bioorg. Med. Chem..

[28] Minotti G., Menna P., Salvatorelli E., Cairo G., Gianni L. (2004). Anthracyclines: Molecular Advances and Pharmacologie Developments in Antitumor Activity and Cardiotoxicity. Pharmacol. Rev..

[29] Beckford S.J., Dixon D.W. (2012). Molecular Dynamics of Anthraquinone DNA Intercalators with Polyethylene Glycol Side Chains. J. Biomol. Struct. Dyn..

[30] Mikaelian G., Megariotis G., Theodorou D.N. (2024). Interactions of a Novel Anthracycline with Oligonucleotide DNA and Cyclodextrins in an Aqueous Environment. J. Phys. Chem. B.

[31] Xie J., Zhang J., Chen X. (2025). Aloe-Emodin: From Pharmacological Mechanisms to Clinical Applications and Future Perspectives. Front. Pharmacol..

[32] Yu C.P., Shia C.S., Lin H.J., Hsieh Y.W., Lin S.P., Hou Y.C. (2016). Analysis of the Pharmacokinetics and Metabolism of Aloe-Emodin Following Intravenous and Oral Administrations in Rats. Biomed. Chromatogr..

[33] Duan H.G., Wei Y.H., Li B.X., Qin H.Y., Wu X.A. (2009). Improving the Dissolution and Oral Bioavailability of the Poorly Water-Soluble Drug Aloe-Emodin by Solid Dispersion with Polyethylene Glycol 6000. Drug Dev. Res..

[34] Semerel J., John N., Fardim P., Dehaen W. (2026). Recent Developments in Chemical Synthesis and Biological Activities of Aloe-Emodin Derivatives. Organics.

[35] Kumar G.D., Siva B., Vadlamudi S., Bathula S.R., Dutta H., Suresh Babu K. (2021). Design, Synthesis, and Biological Evaluation of Pyrazole-Linked Aloe Emodin Derivatives as Potential Anticancer Agents. RSC Med. Chem..

[36] Dileep Kumar G., Siva B., Ashwini K., Vinod Kumar J., Ramalingam V., Sai Balaji A., Suresh Babu K. (2023). Design, Synthesis, Cytotoxic, and Anti-Inflammatory Activities of Some Novel Analogues of Aloe-Emodin Isolated from the Rhizomes of Rheum Emodi. Nat. Prod. Res..

[37] Rixson J.E., Abraham J.R., Egoshi Y., Skelton B.W., Young K., Gilbert J., Sakoff J.A., Gericke K.M., McCluskey A., Stewart S.G. (2015). The Synthesis and Biological Activity of Novel Anthracenone-Pyranones and Anthracenone-Furans. Bioorg. Med. Chem..

[38] Tikhomirov A.S., Shchekotikhin A.E., Lee Y.H., Chen Y.A., Yeh C.A., Tatarskiy V.V., Dezhenkova L.G., Glazunova V.A., Balzarini J., Shtil A.A. (2015). Synthesis and Characterization of 4,11-Diaminoanthra[2,3-b]Furan-5,10-Diones: Tumor Cell Apoptosis through TNOX-Modulated NAD+/NADH Ratio and SIRT1. J. Med. Chem..

[39] Shchekotikhin A.E., Dezhenkova L.G., Tsvetkov V.B., Luzikov Y.N., Volodina Y.L., Tatarskiy V.V., Kalinina A.A., Treshalin M.I., Treshalina H.M., Romanenko V.I. (2016). Discovery of Antitumor Anthra[2,3-b]Furan-3-Carboxamides: Optimization of Synthesis and Evaluation of Antitumor Properties. Eur. J. Med. Chem..

[40] Tikhomirov A.S., Lin C.Y., Volodina Y.L., Dezhenkova L.G., Tatarskiy V.V., Schols D., Shtil A.A., Kaur P., Chueh P.J., Shchekotikhin A.E. (2018). New Antitumor Anthra[2,3-b]Furan-3-Carboxamides: Synthesis and Structure-Activity Relationship. Eur. J. Med. Chem..

[41] Horvat M., Avbelj M., Durán-Alonso M.B., Banjanac M., Petković H., Iskra J. (2021). Antiviral Activities of Halogenated Emodin Derivatives against Human Coronavirus NL63. Molecules.

[42] Bringmann G., Menche D., Kraus J., Mühlbacher J., Peters K., Peters E.M., Brun R., Bezabih M., Abegaz B.M. (2002). Atropo-Enantioselective Total Synthesis of Knipholone and Related Antiplasmodial Phenylanthraquinones. J. Org. Chem..

[43] Mzhelskaya M.A., Ivanchikova I.D., Polyakov N.E., Moroz A.A., Shvartsberg M.S. (2004). Cyclization of 1-Hydroxy-2-(Oxoalkynyl)Anthraquinones. Russ. Chem. Bull..

[44] Liang X.Y., Battini N., Sui Y.F., Ansari M.F., Gan L.L., Zhou C.H. (2021). Aloe-Emodin Derived Azoles as a New Structural Type of Potential Antibacterial Agents: Design, Synthesis, and Evaluation of the Action on Membrane, DNA, and MRSA DNA Isomerase. RSC Med. Chem..

[45] Deng Z., Bheemanaboina R.R.Y., Luo Y., Zhou C.H. (2022). Aloe Emodin-Conjugated Sulfonyl Hydrazones as Novel Type of Antibacterial Modulators against S. Aureus 25923 through Multifaceted Synergistic Effects. Bioorg. Chem..

[46] Wang Y.X., Deng Z., Bibi A., Fang B., Zhou C.H. (2024). Unique Azolyl Acylhydrazonyl Hybridization of Aloe Emodins to Access Potential Antibacterial Agents. Chin. J. Chem..

[47] Di L., Kerns E.H. (2006). Biological Assay Challenges from Compound Solubility: Strategies for Bioassay Optimization. Drug Discov. Today.

[48] Alexander J., Bhatia A.V., Mitscher L.A., Omoto S., Suzuki T. (1980). Methylation and Hydroxylation Studies on Aloe-Emodin. J. Org. Chem..

