# Supplementary material for: Novel Aloe-Emodin Derivatives as Potential Anticancer Agents: Synthesis, Characterization and Cytotoxic Activity
Source: Molecules. 2026 May 15;31(10):1676. doi: 10.3390/molecules31101676 (PMC13210113; doi:10.3390/molecules31101676)
Supplement: Supplementary file 1 [file molecules-31-01676-s001.zip › molecules-4269278-supplementary.pdf]

Novel Aloe-Emodin Derivatives as Potential Anticancer Agents: Synthesis, Characterization and Cytotoxic Activity

Jeltzlin Semerel [1], Shuhe Zheng [2], Haoyue Hu [3], Yuyu Fang [3], Nigel John [1], Pedro Fardim [4] and Wim Dehaen [5,*]

[1] SISSTEM Program, Faculty of Arts and Science, University of Aruba, J. Irausquinplein 4, Oranjestad, Aruba; jeltzlin.semerel@ua.aw (J.S.); nigel.john@ua.aw (N.J.)
[2] Institut de Science et D'ingénierie Supramoléculaires, University of Strasbourg, 8 Allée Gaspard Monge, 67000 Strasbourg, France; shuhe.zheng@ics-cnrs.unistra.fr
[3] School of Pharmacy, Chengdu University of Traditional Chinese Medicine, Chengdu 611137, China; huhaoyue@stu.cdutcm.edu.cn (H.H.); yyfang@cdutcm.edu.cn (Y.F.)
[4] Department of Chemical Engineering, KU Leuven, Celestijnenlaan 200F, B-3001 Leuven, Belgium; pedro.fardim@kuleuven.be
[5] Department of Chemistry, KU Leuven, Celestijnenlaan 200F, B-3001 Leuven, Belgium
* Correspondence: wim.dehaen@kuleuven.be

NMR spectra

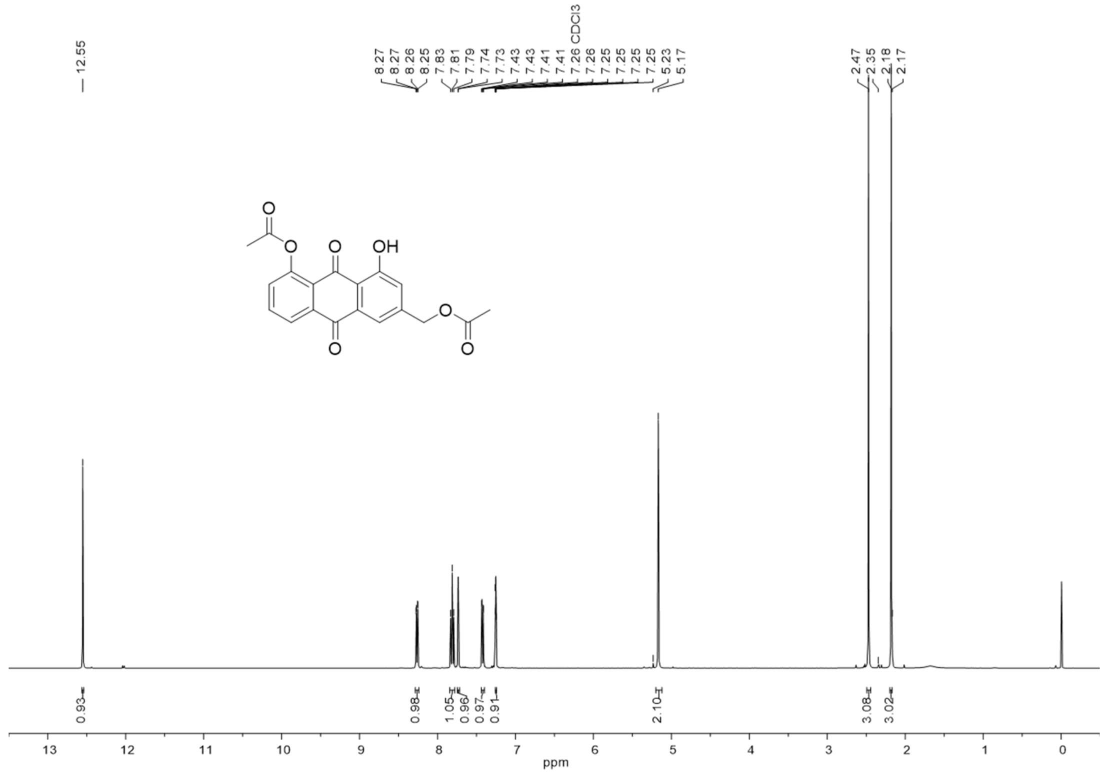

Figure S1: ^{1}H, 400 Hz, $CDCl_3$, **2**

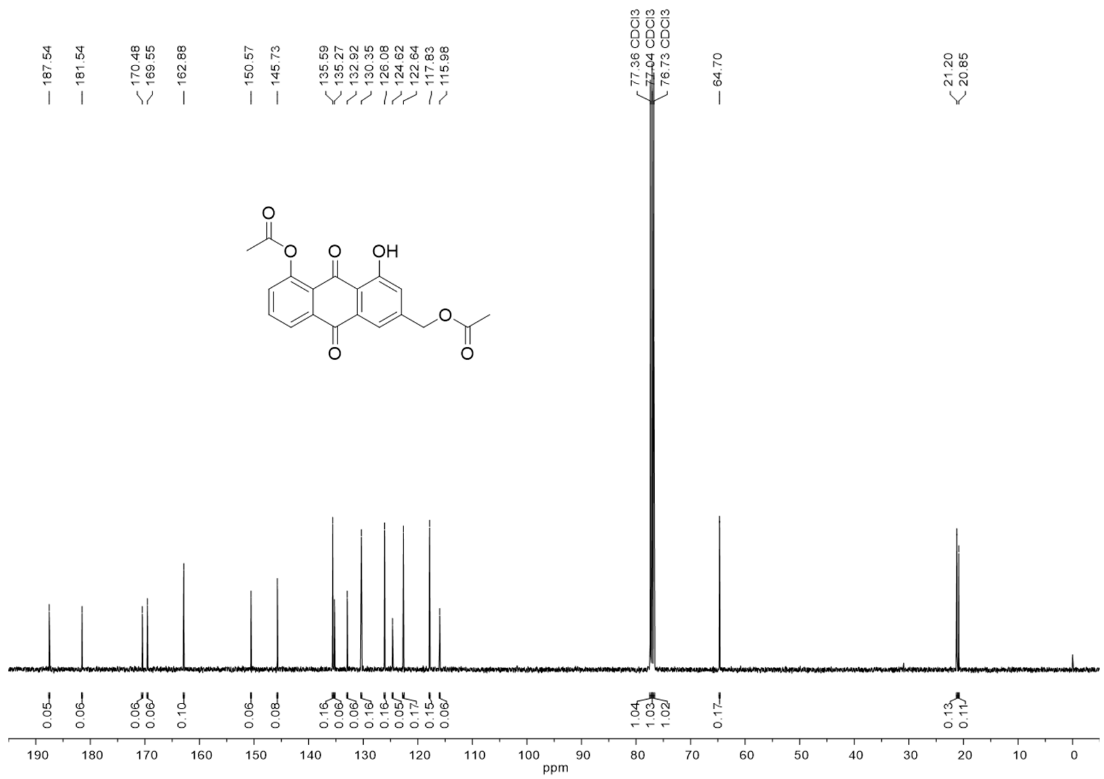

Figure S2: ^{13}C, 101 MHz, $CDCl_3$, **2**

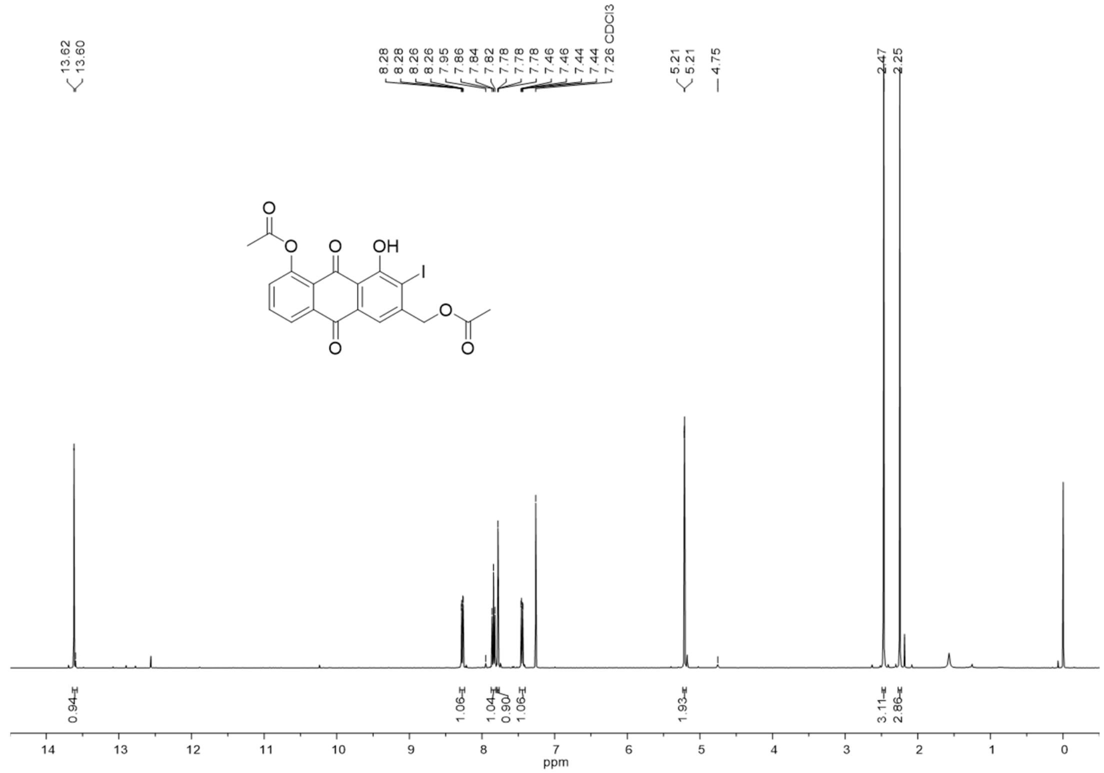

Figure S3: ^{1}H, 400 Hz, $CDCl_3$, **3**

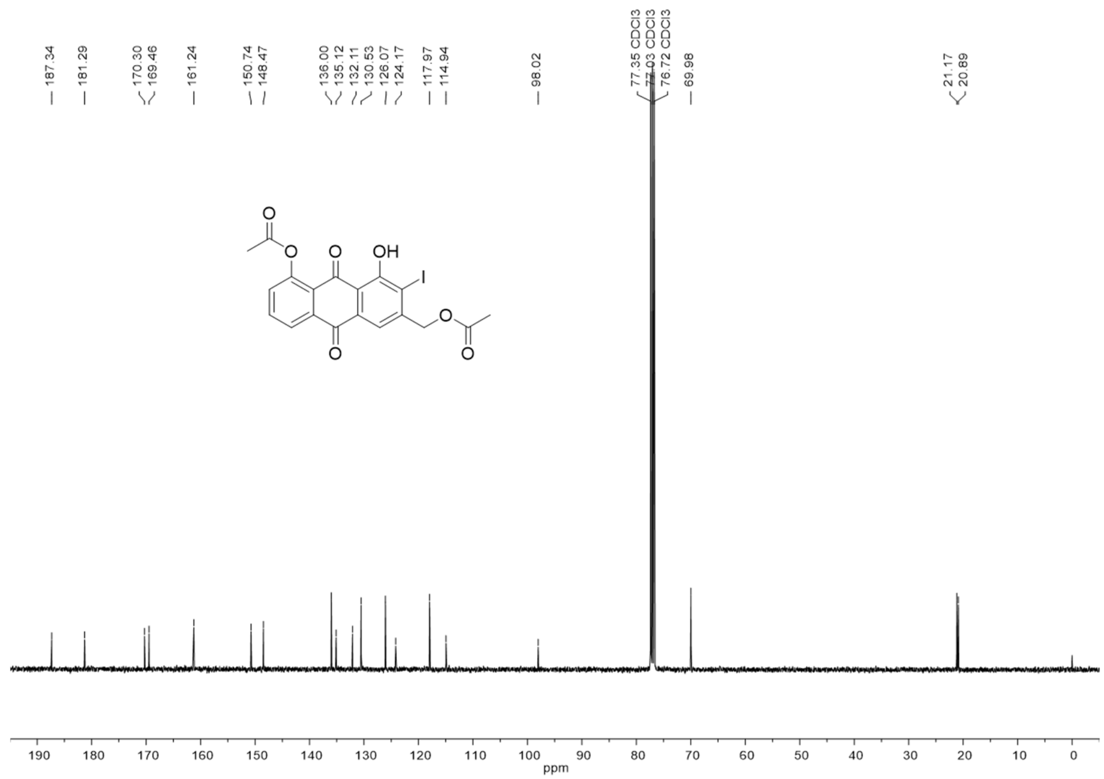

Figure S4: ^{13}C, 101 MHz, $CDCl_3$, **3**

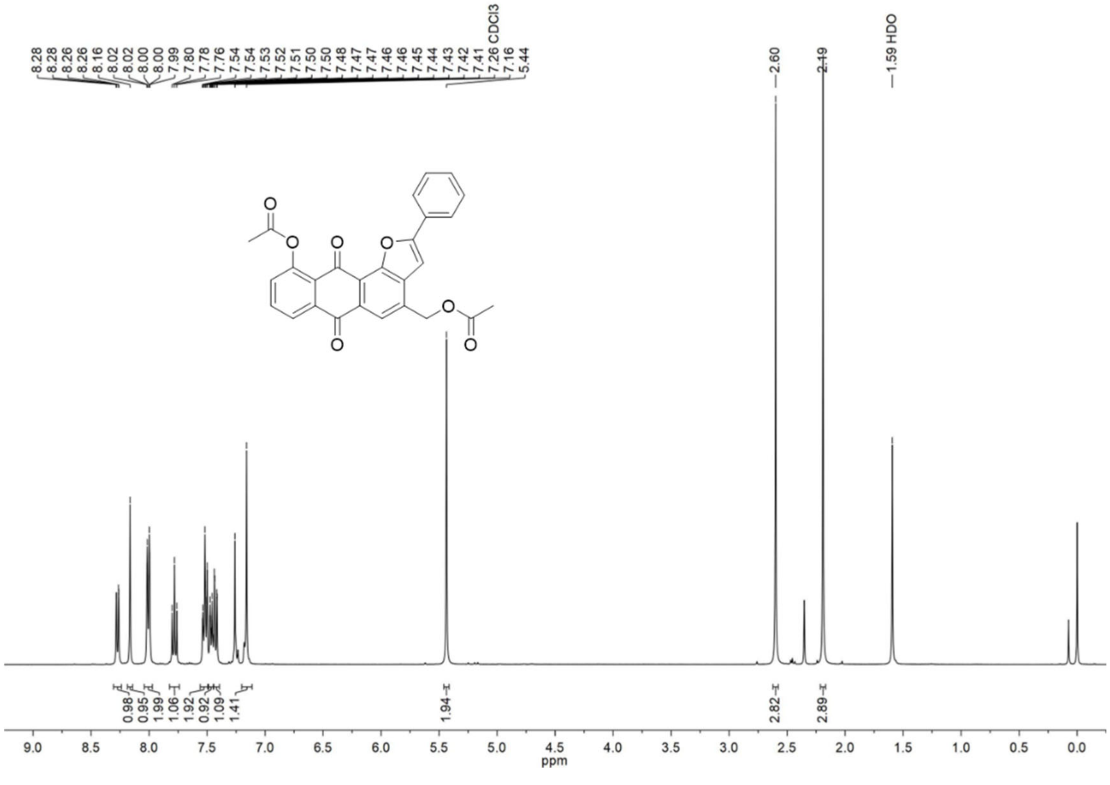

Figure S5: ^{1}H, 400 Hz, $CDCl_3$, **5a**

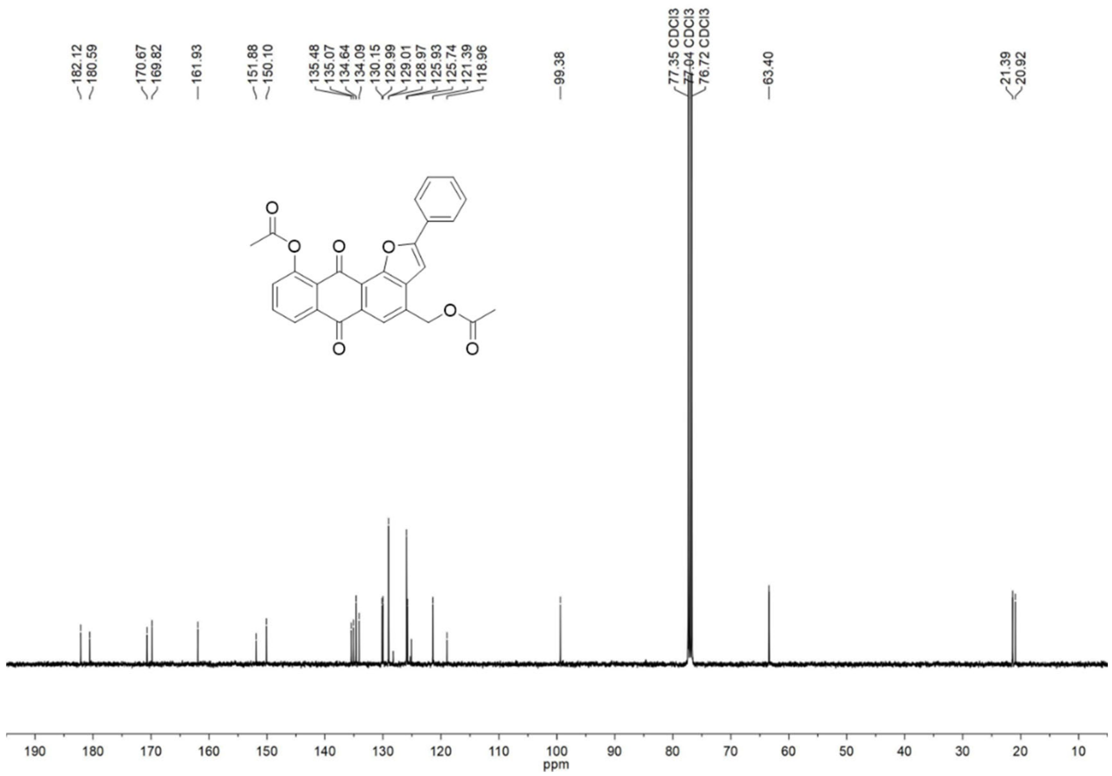

Figure S6: ^{13}C, 101 MHz, $CDCl_3$, **5a**

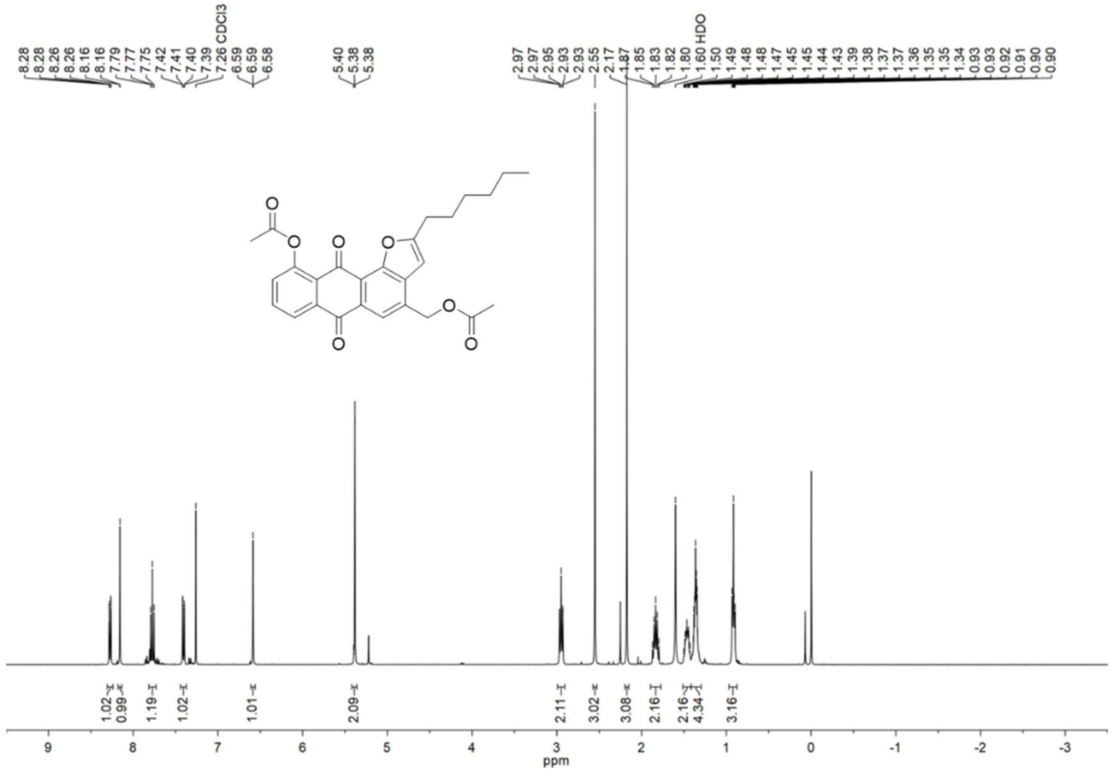

Figure S7: ^{1}H, 400 Hz, $CDCl_3$, **5b**

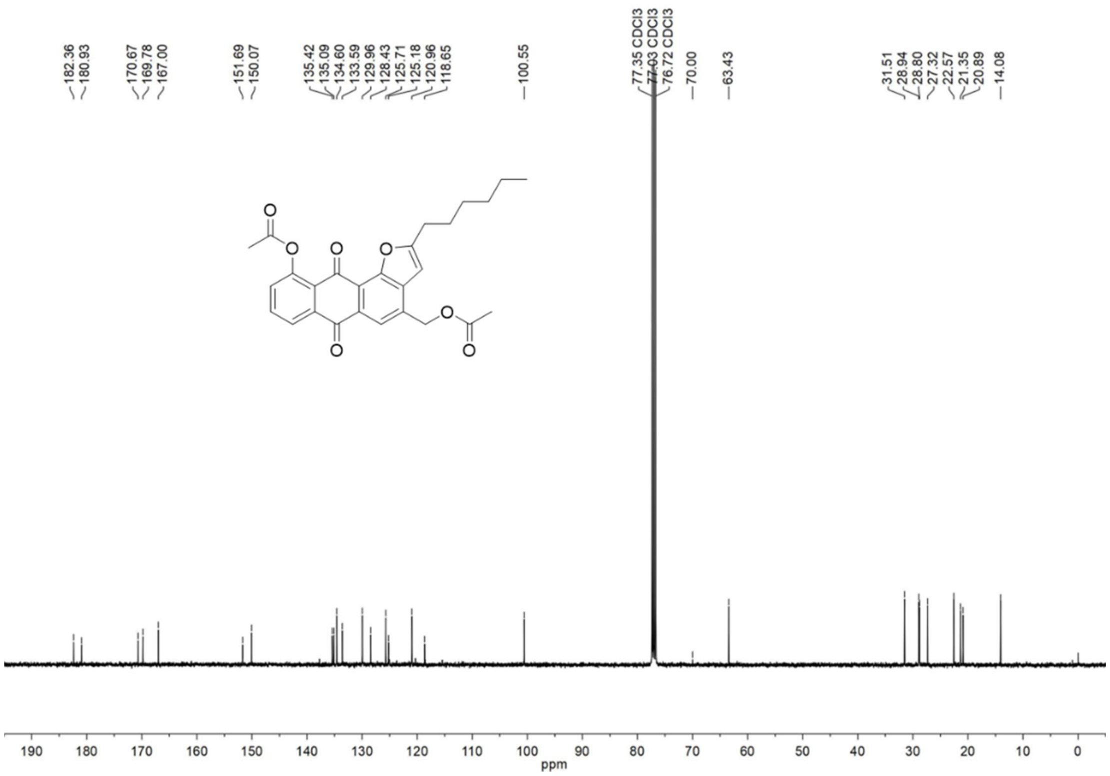

Figure S8: ^{13}C, 101 MHz, $CDCl_3$, **5b**

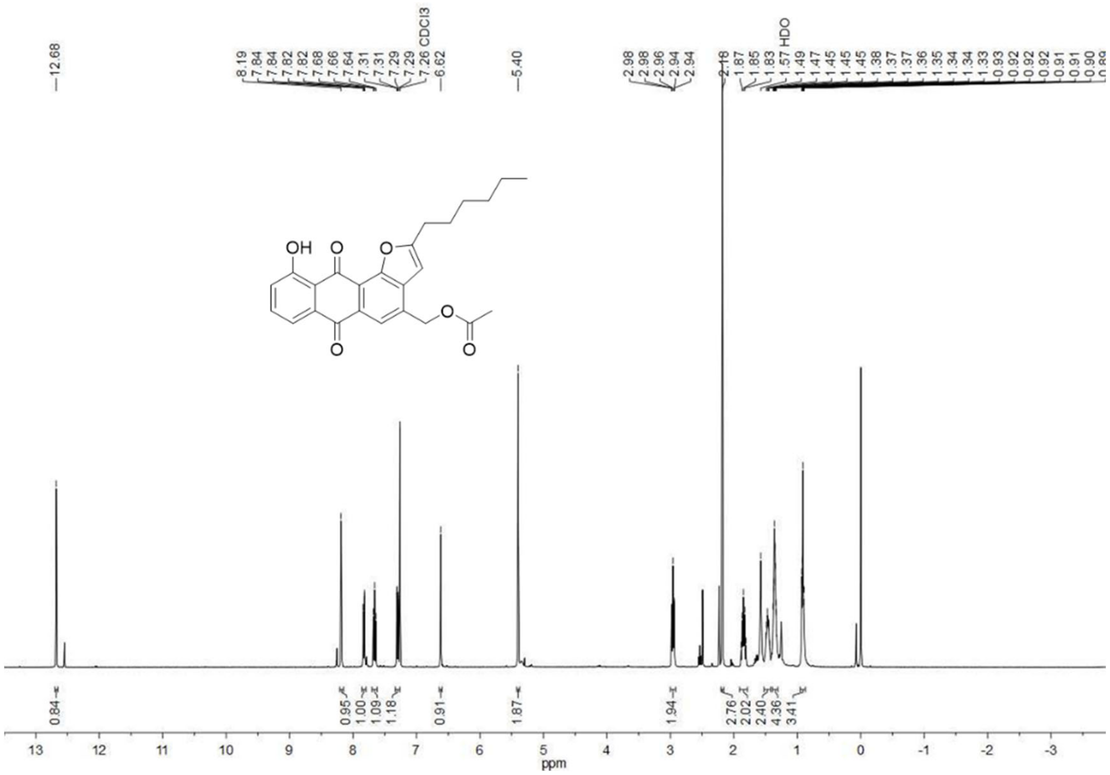

Figure S9: ^{1}H, 400 Hz, $CDCl_3$, **5c**

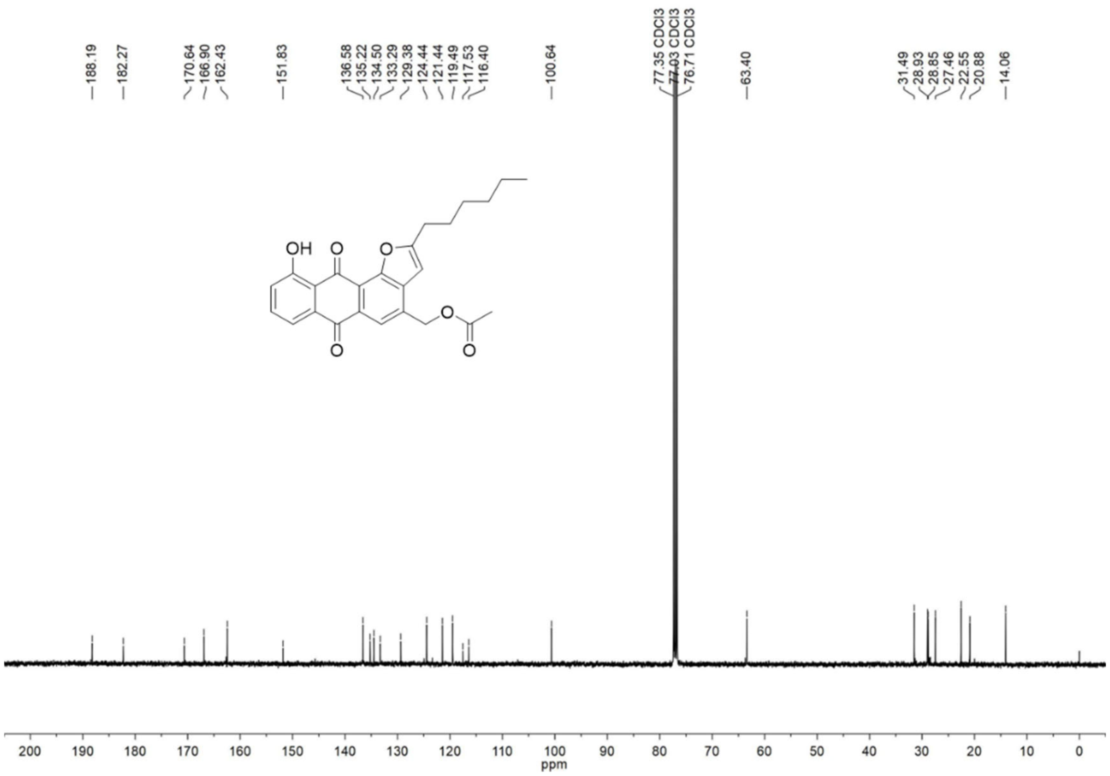

Figure S10: ^{13}C, 101 MHz, $CDCl_3$, **5c**

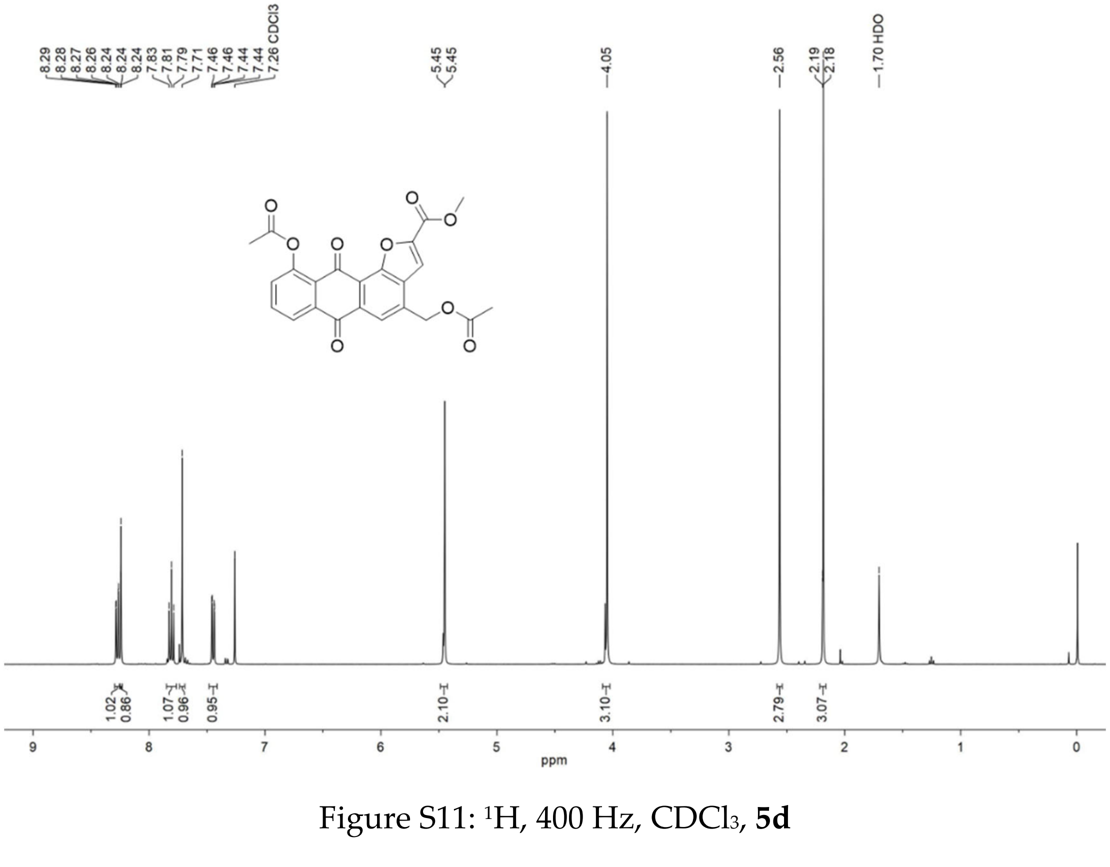

Figure S11: ^{1}H, 400 Hz, $CDCl_3$, **5d**

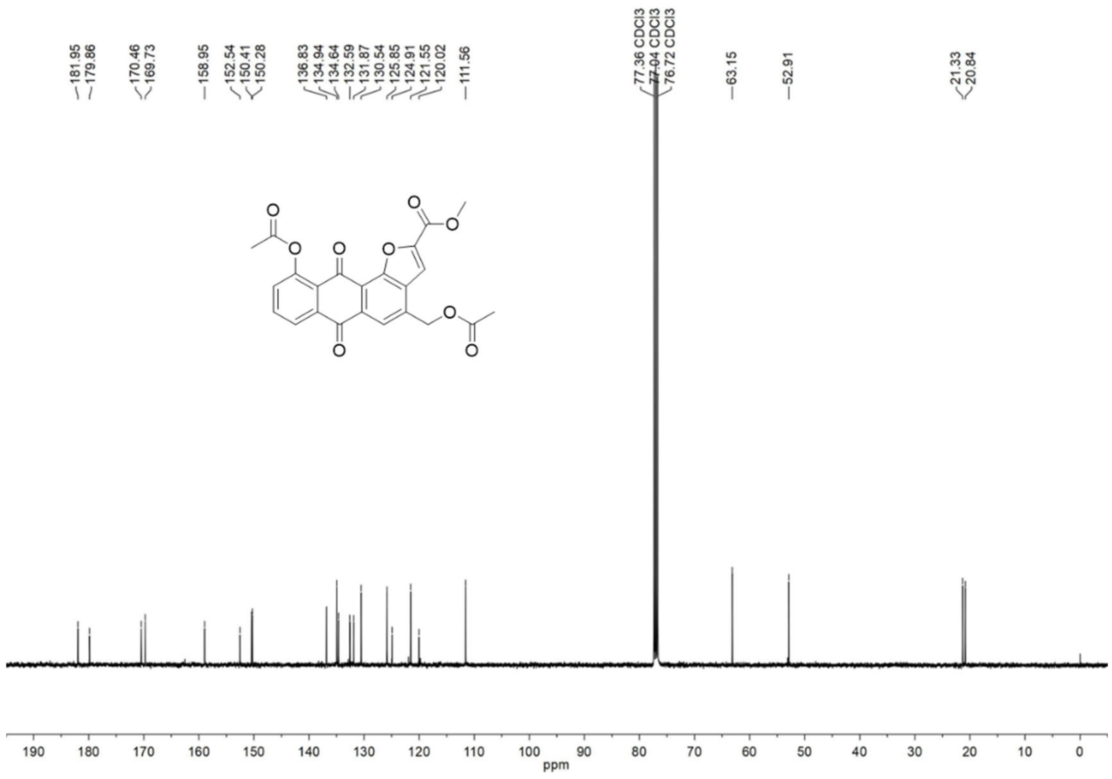

Figure S12: ^{13}C, 101 MHz, $CDCl_3$, **5d**

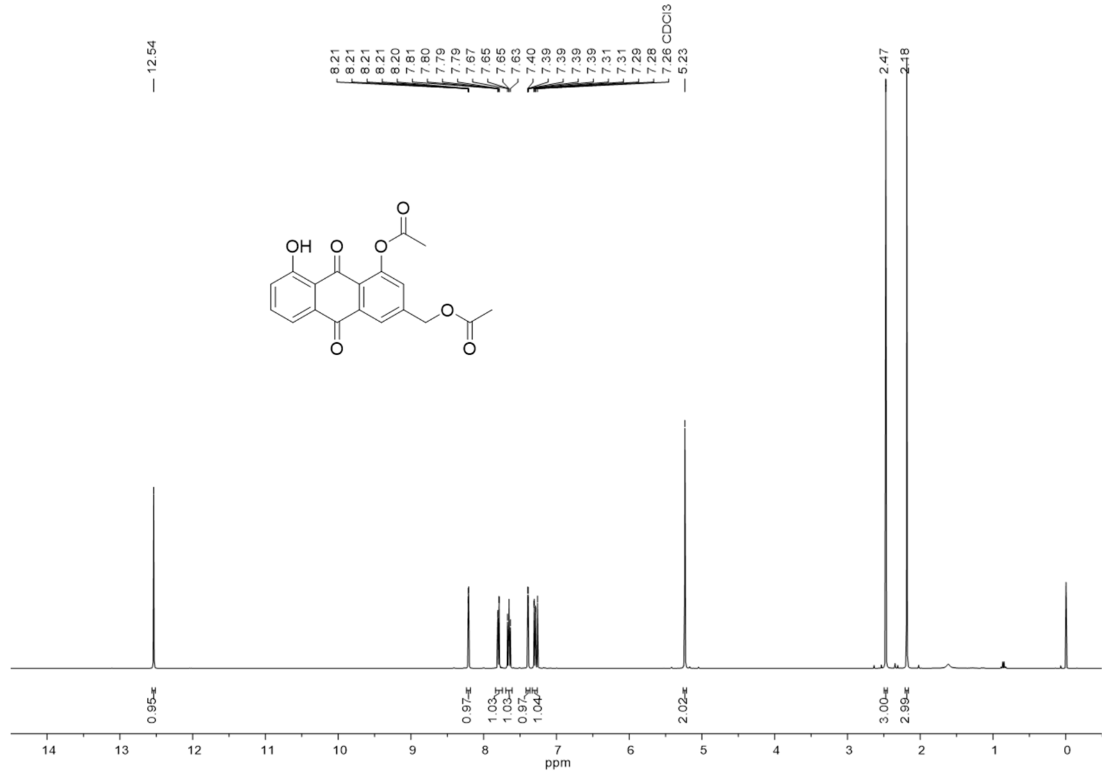

Figure S13: ^{1}H, 400 Hz, $CDCl_3$, **6**

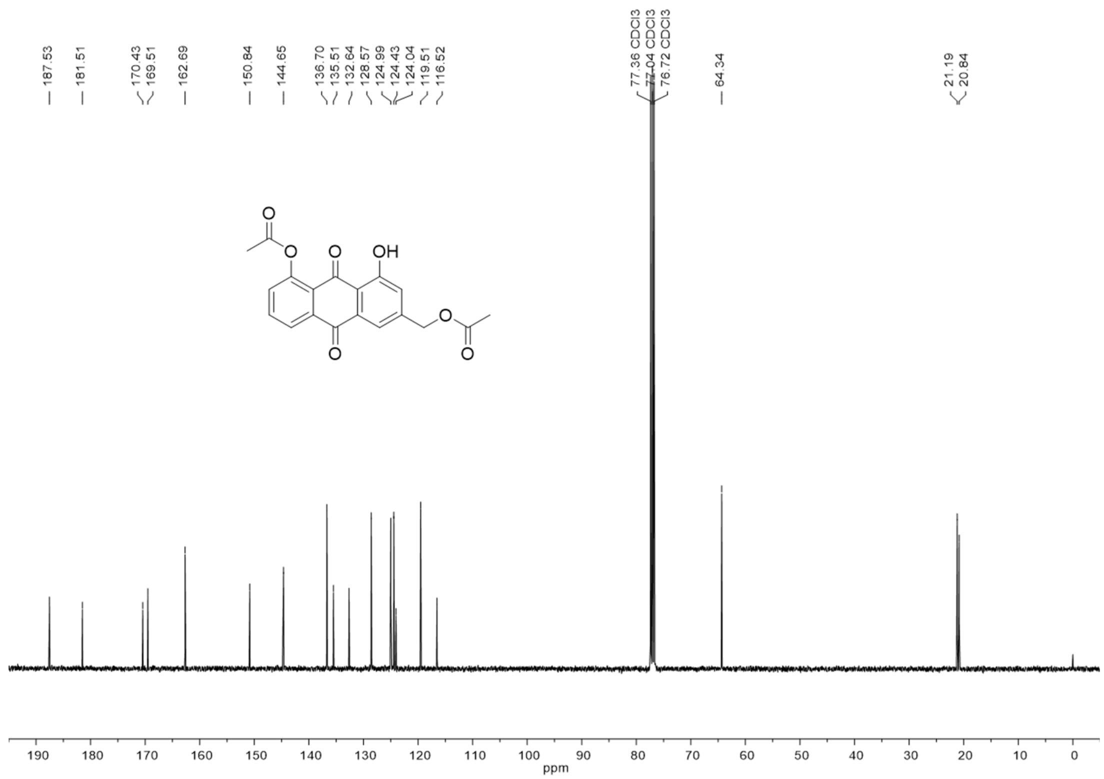

Figure S14: ^{13}C, 101 MHz, $CDCl_3$, **6**

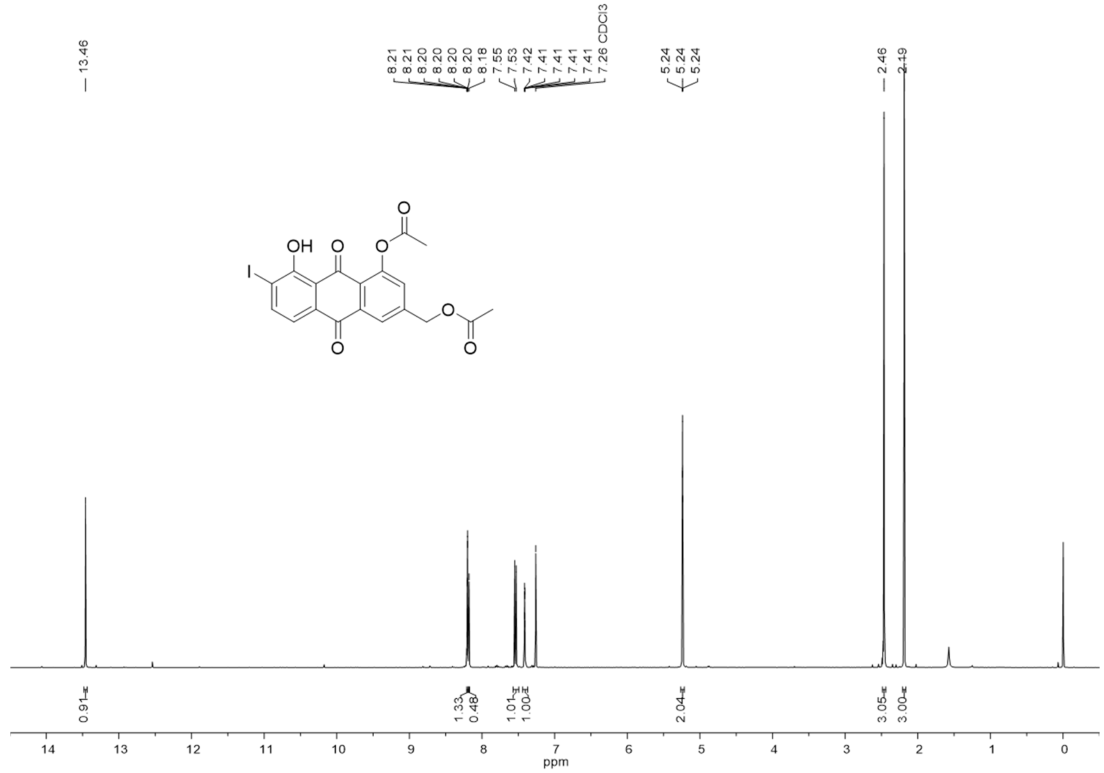

Figure S15: ^{1}H, 400 Hz, $CDCl_3$, **7**

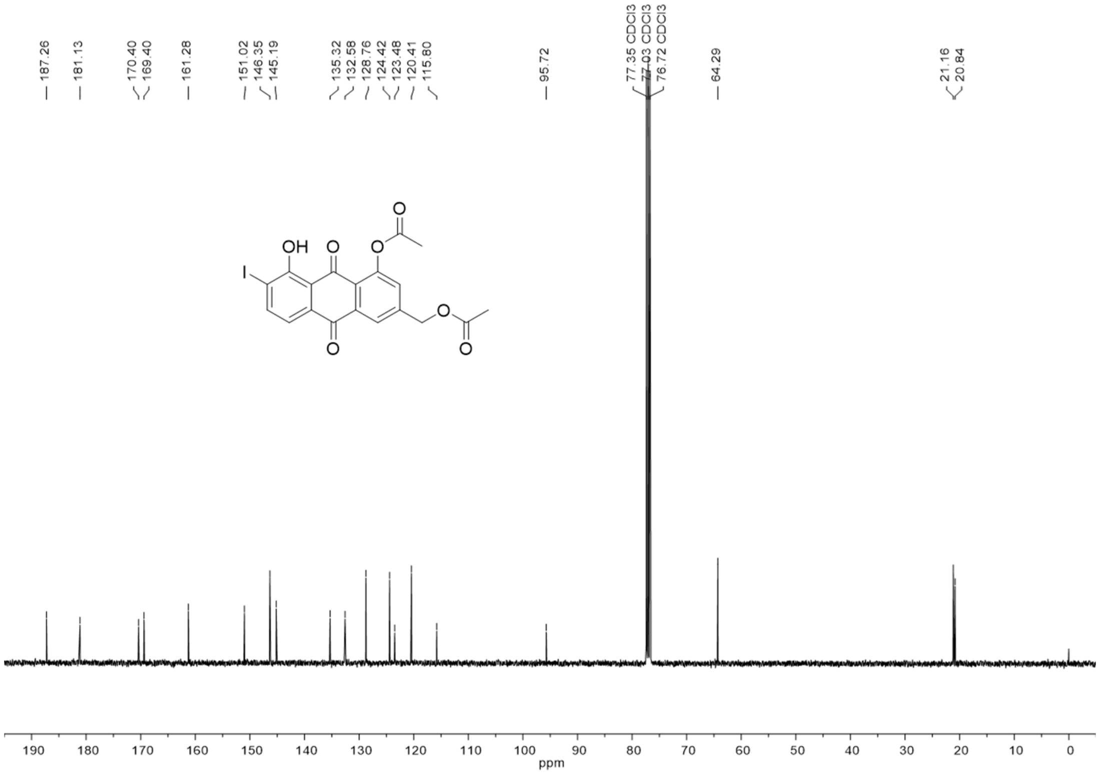

Figure S16: ^{13}C, 101 MHz, $CDCl_3$, **7**

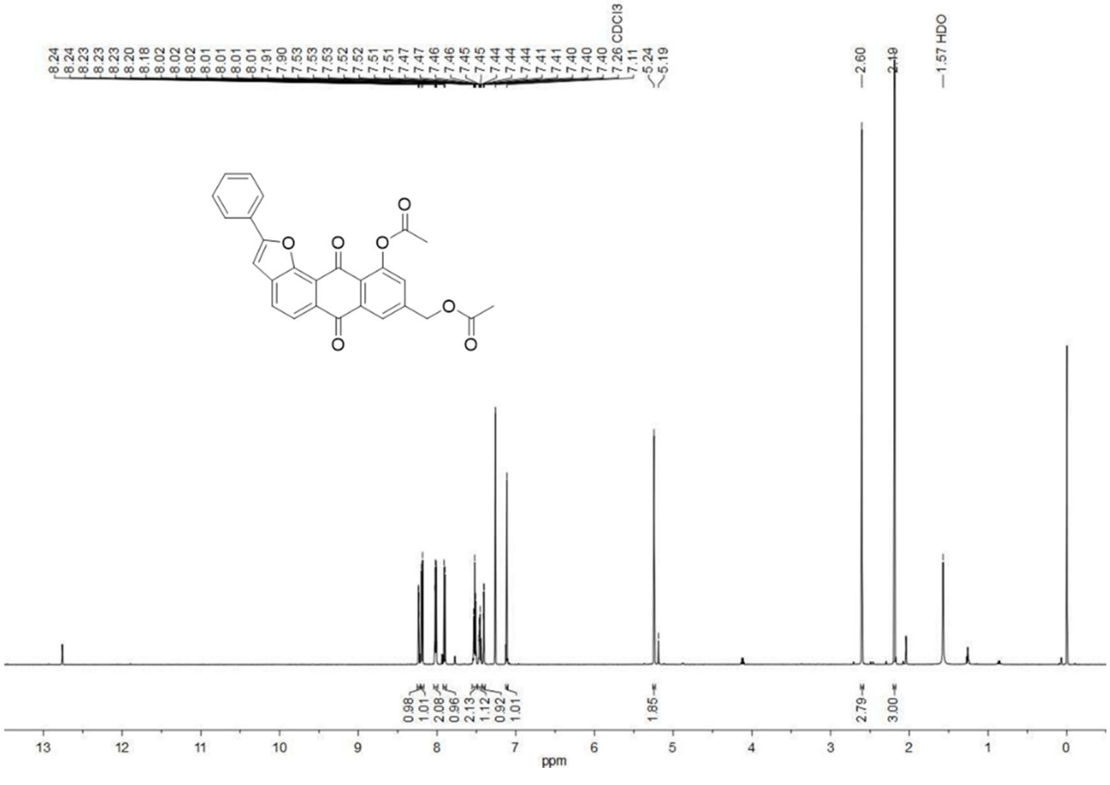

Figure S17: ^{1}H, 400 Hz, $CDCl_3$, **8a**

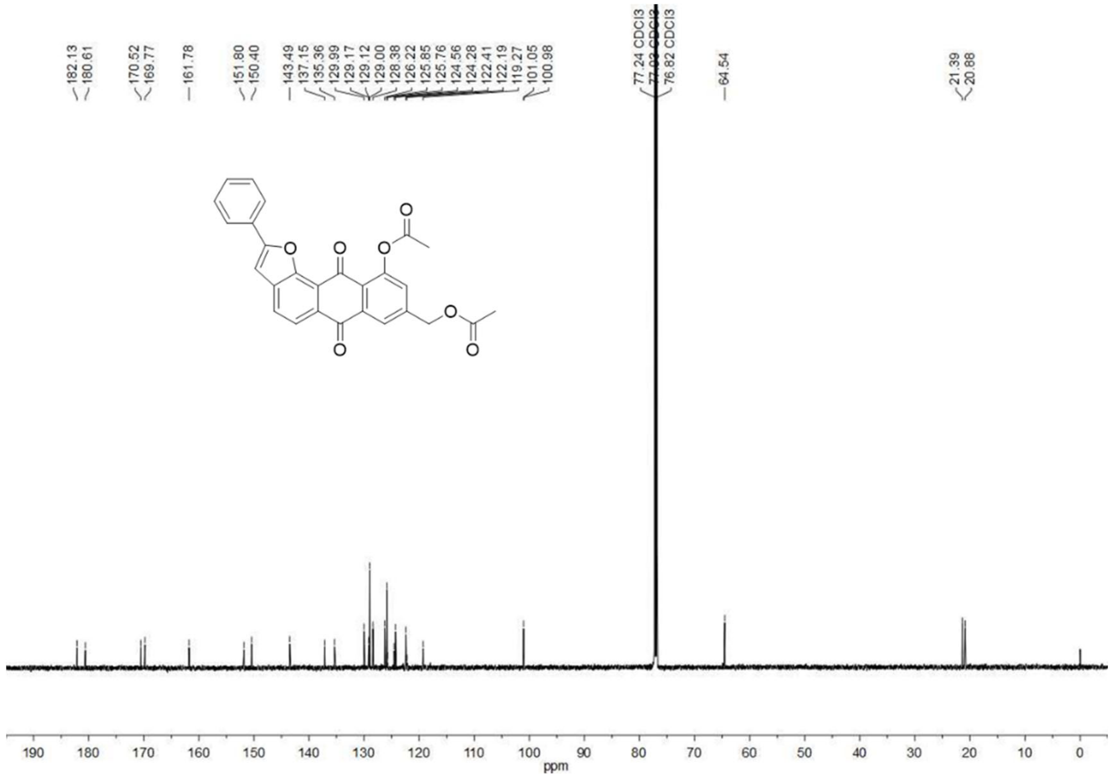

Figure S18: ^{13}C, 101 MHz, $CDCl_3$, **8a**

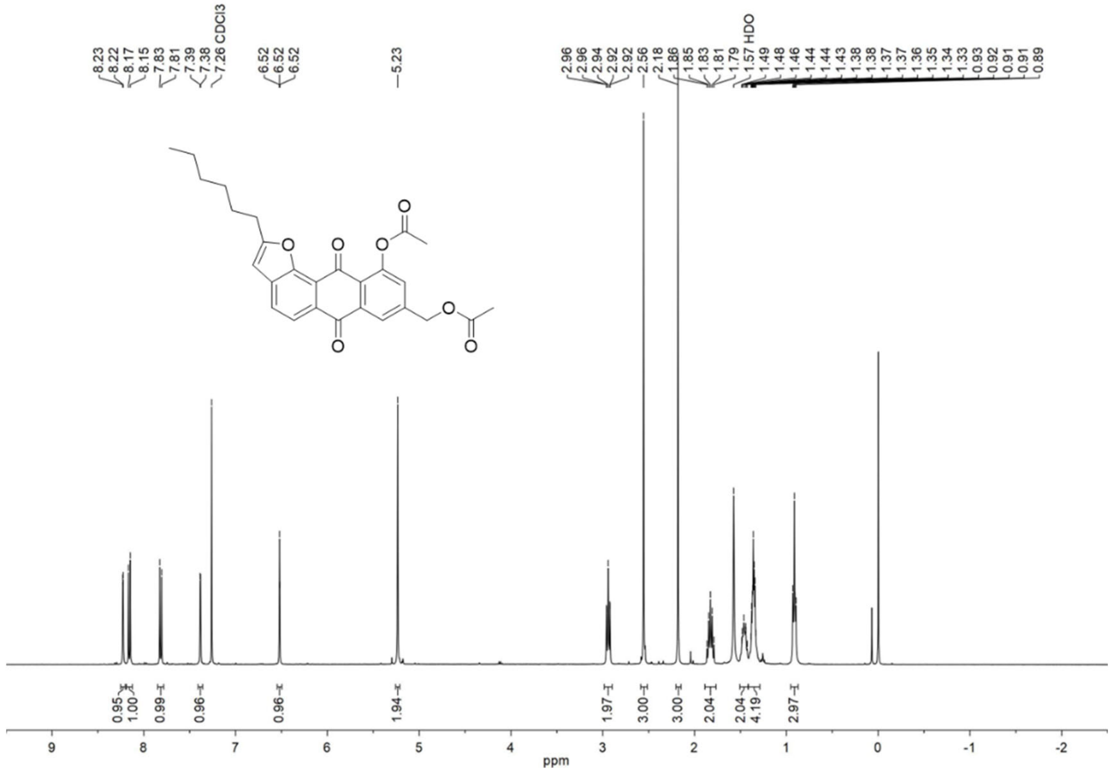

Figure S19: ^{1}H, 400 Hz, $CDCl_3$, **8b**

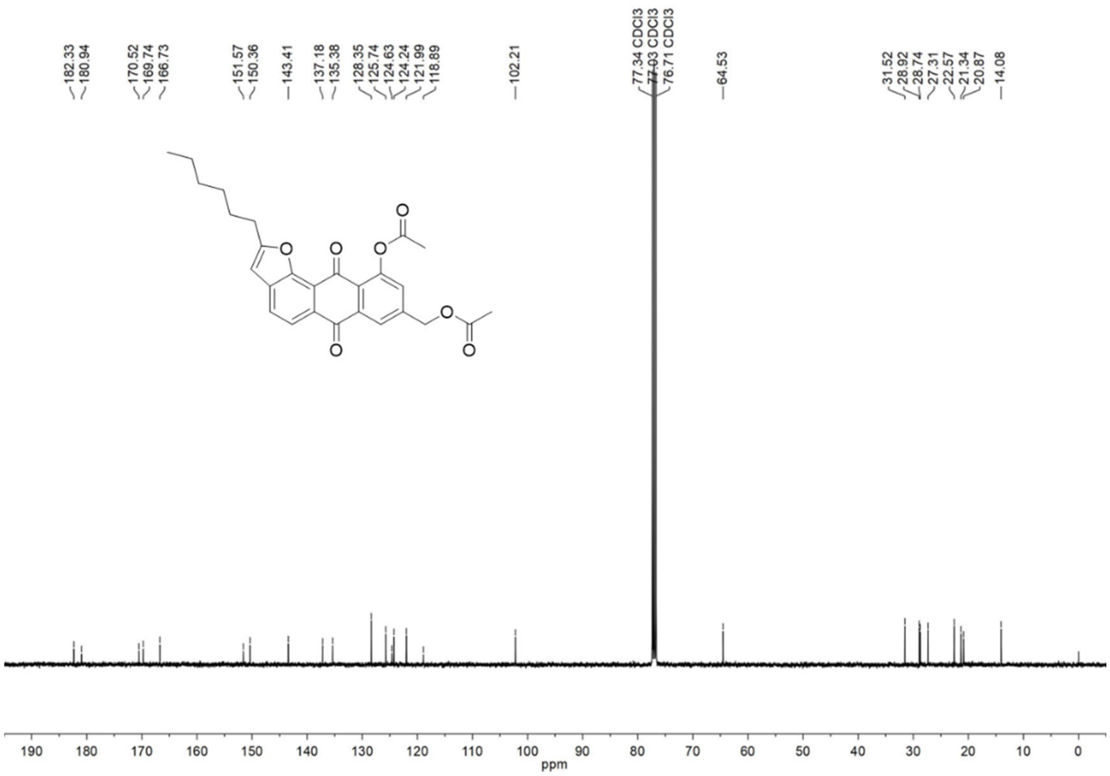

Figure S20: ^{13}C, 101 MHz, $CDCl_3$, **8b**

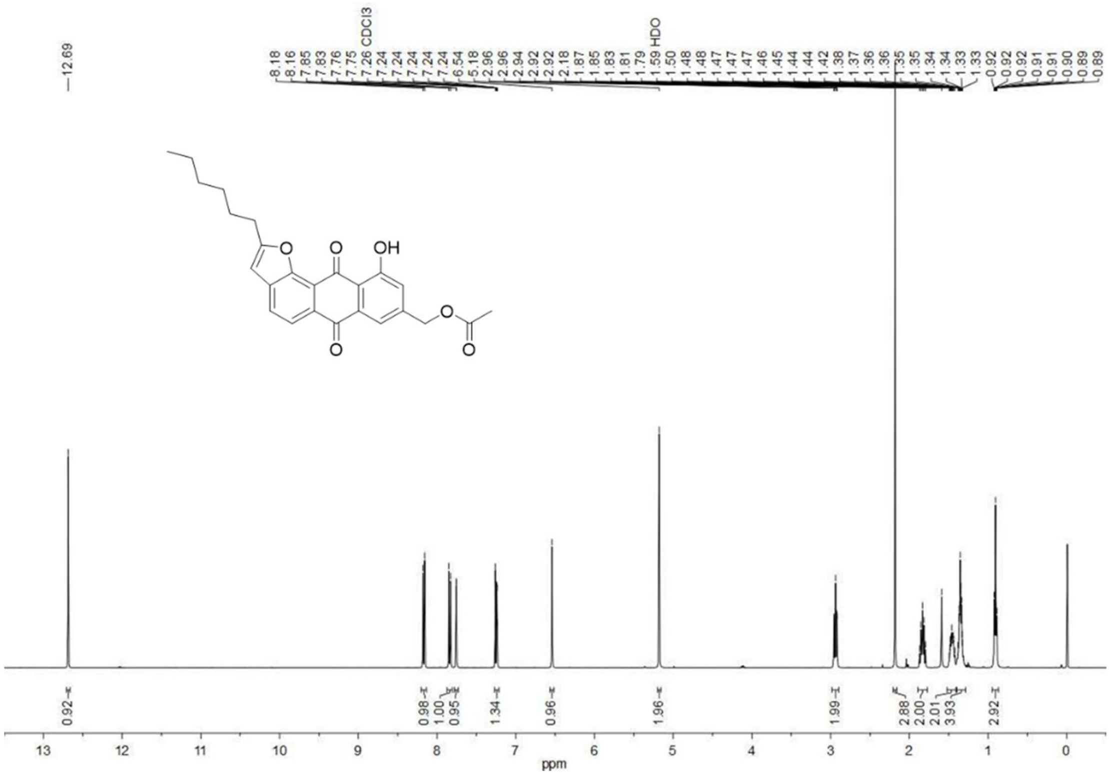

Figure S21: ^{1}H, 400 Hz, $CDCl_3$, **8c**

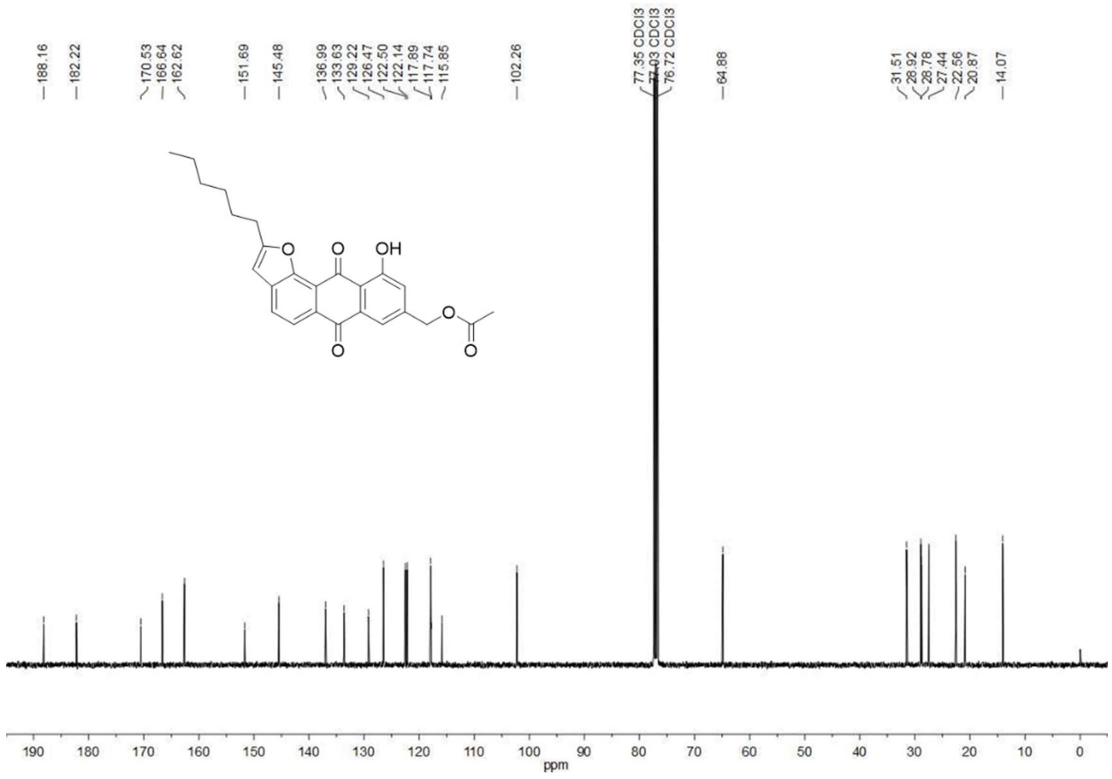

Figure S22: ^{13}C, 101 MHz, $CDCl_3$, **8c**

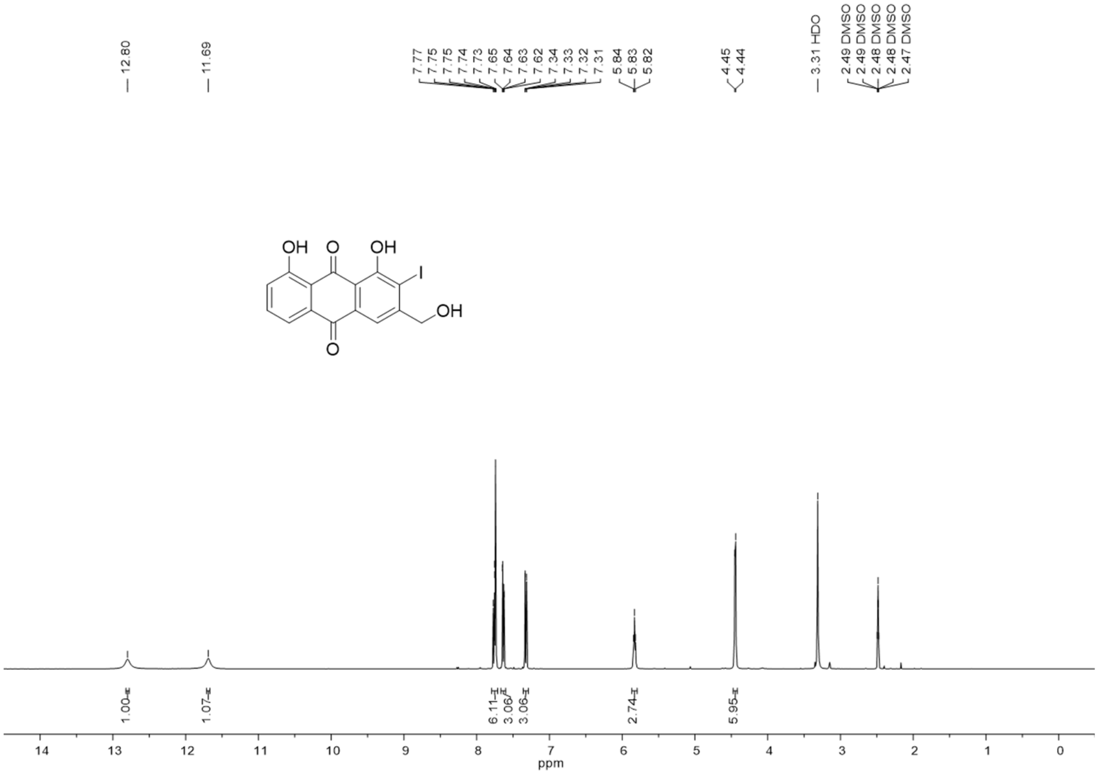

Figure S23: ^{1}H, 400 Hz, DMSO-d_6, **9**

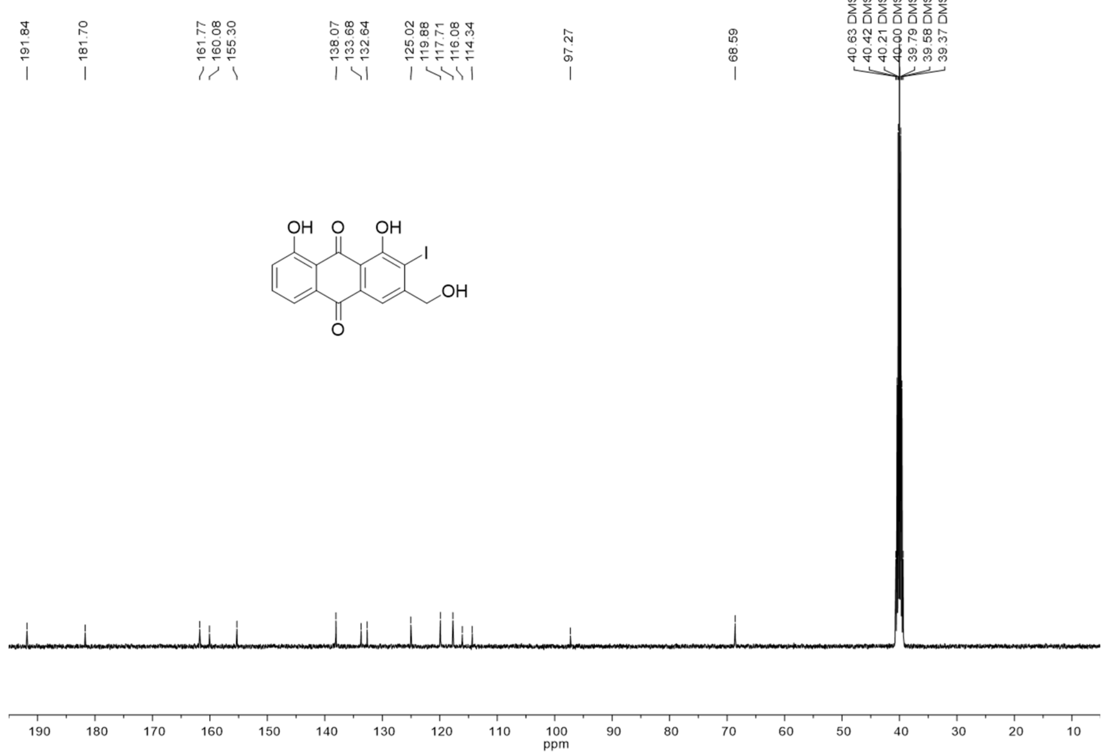

Figure S24: ^{13}C, 101 MHz, DMSO-d_6, **9**

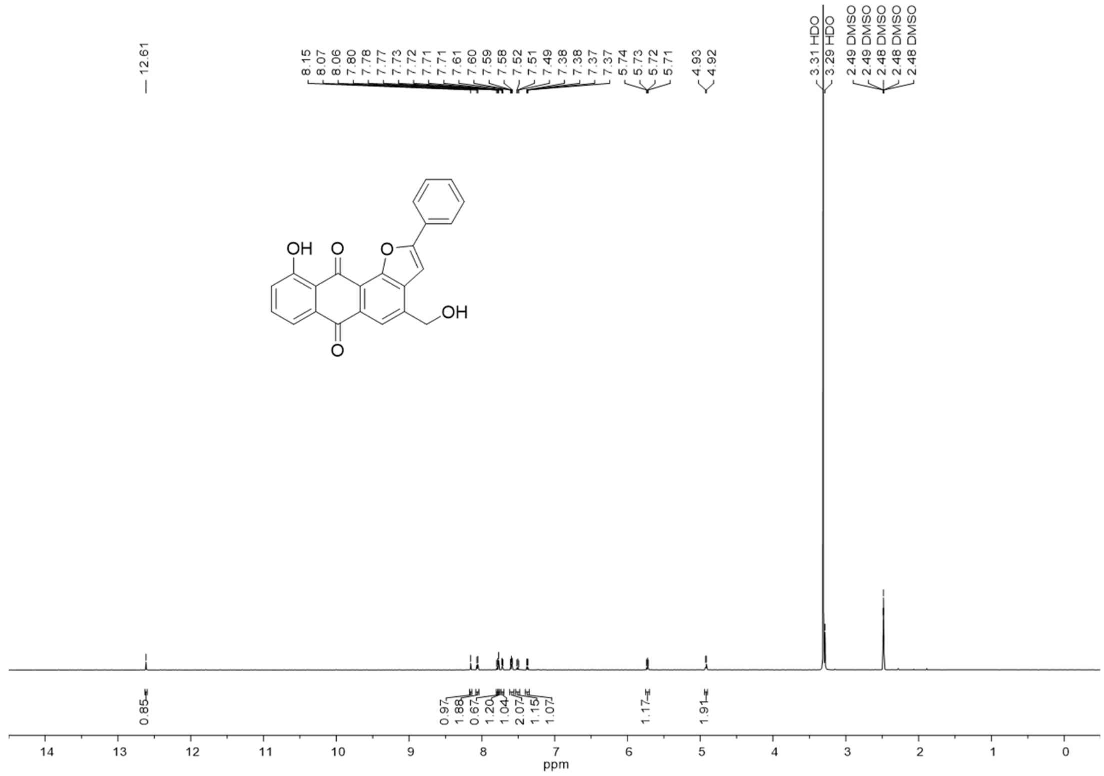

Figure S25: ^{1}H, 400 Hz, DMSO-d_6, **10a**

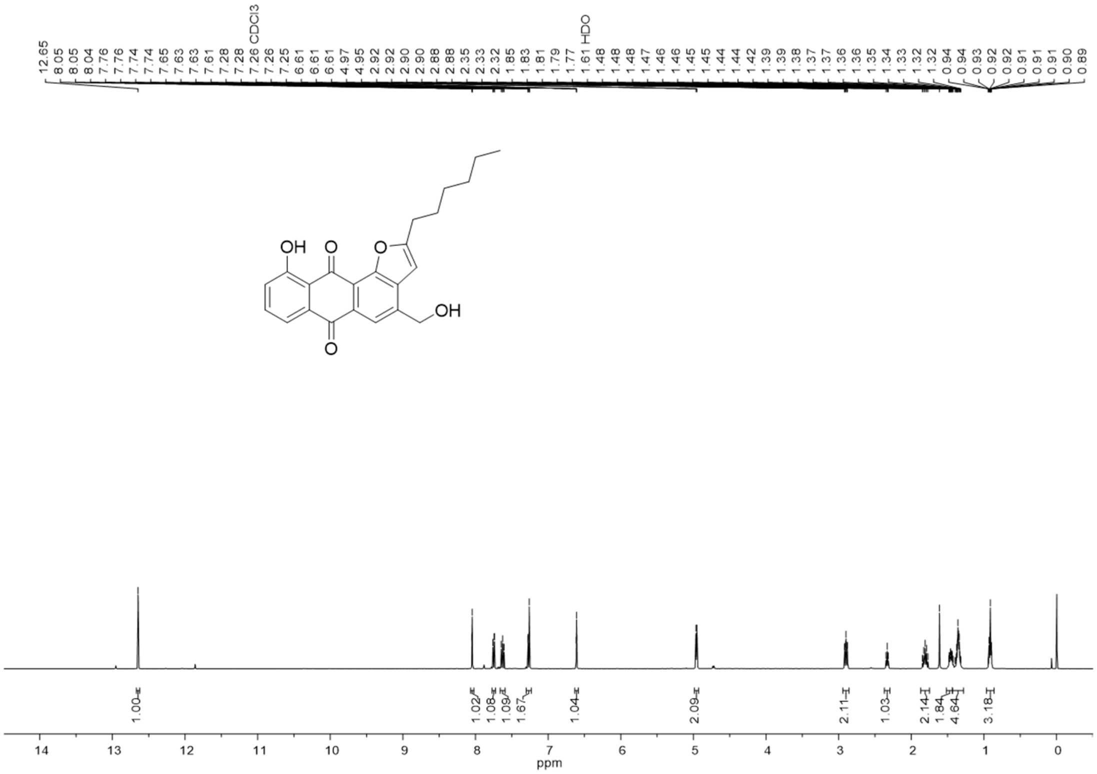

Figure S26: [1]H, 400 Hz, $CDCl_3$, **10b**

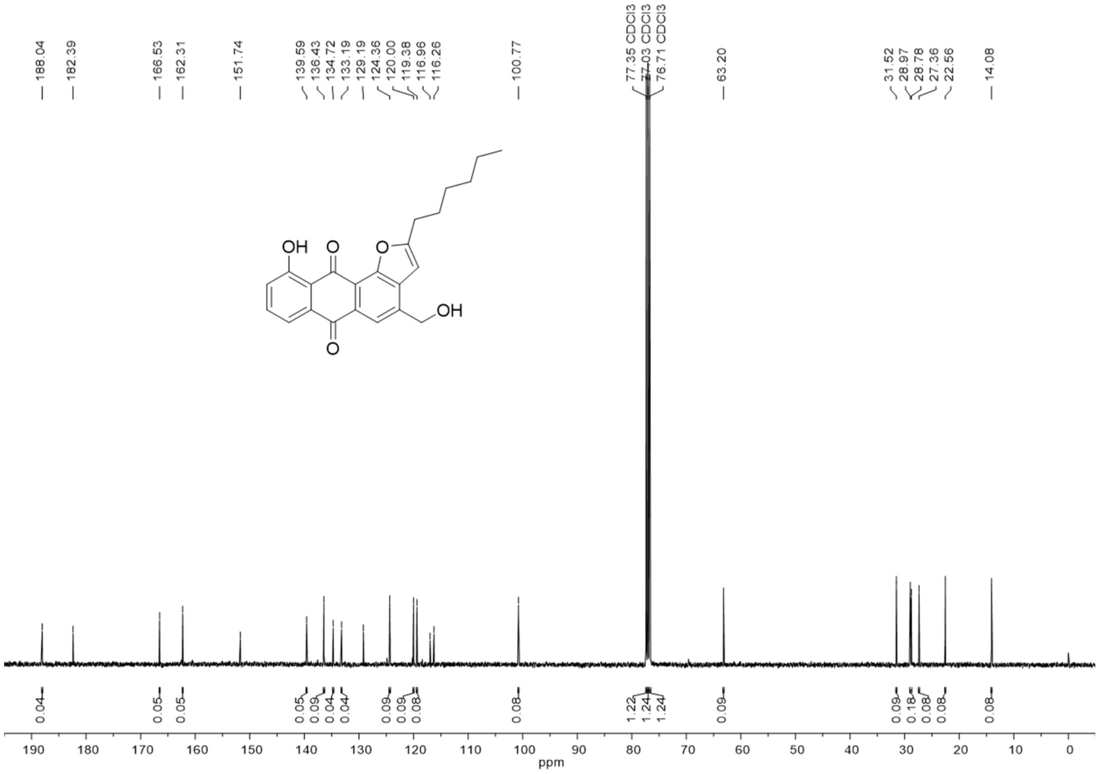

Figure S27: ^{13}C, 101 MHz, $CDCl_3$, **10b**

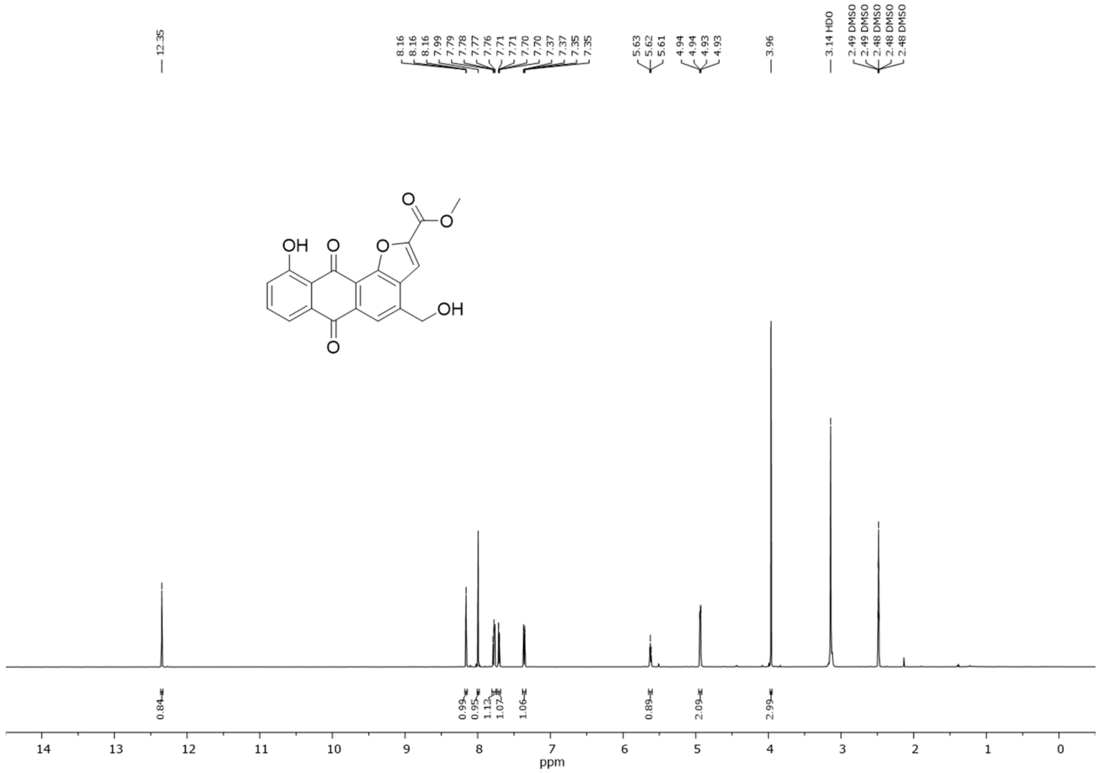

Figure S28: ^{1}H, 600 Hz, 60 ^{o}C, DMSO-d_6, **10c**

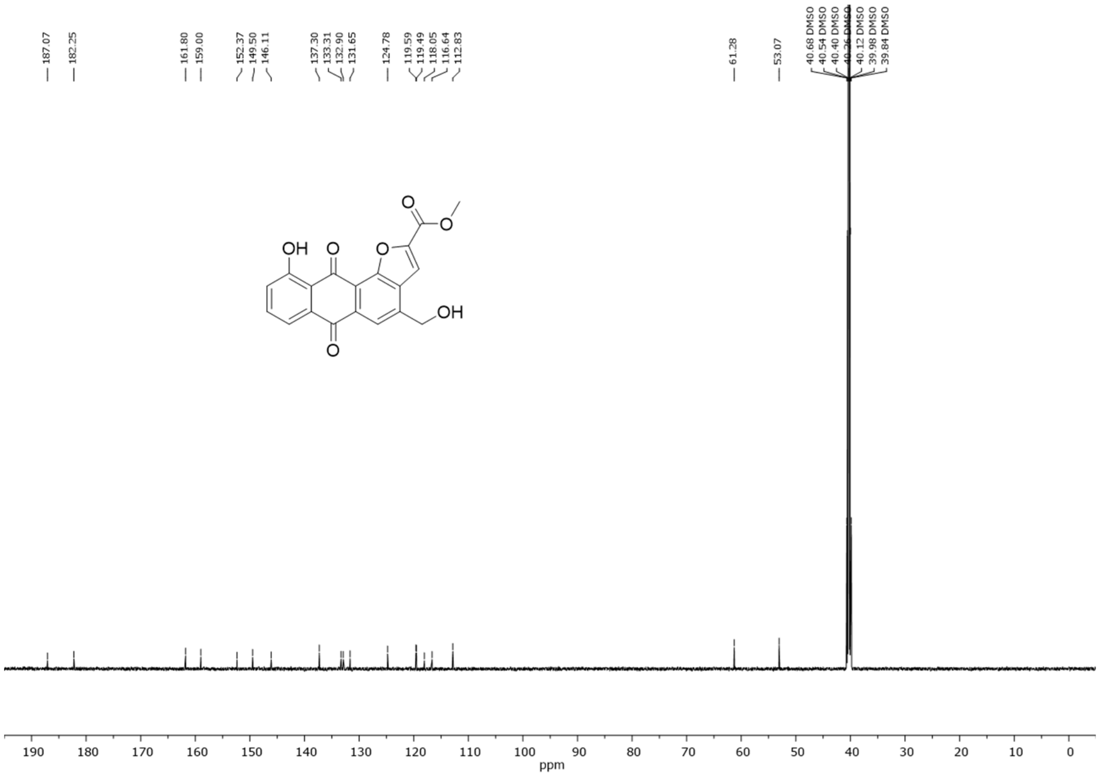

Figure S29: ^{13}C, 151 MHz, 60 ^{o}C, DMSO-d_6, **10c**

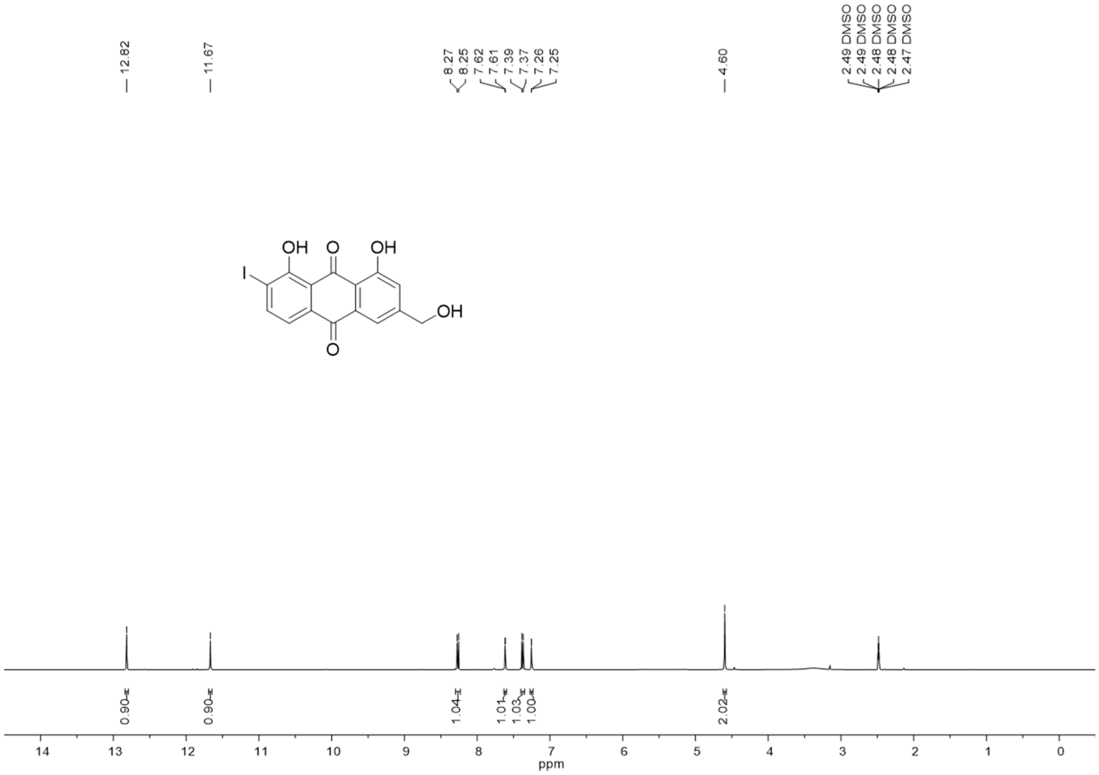

Figure S30: ^{1}H, 400 Hz, DMSO-d_6, **11**

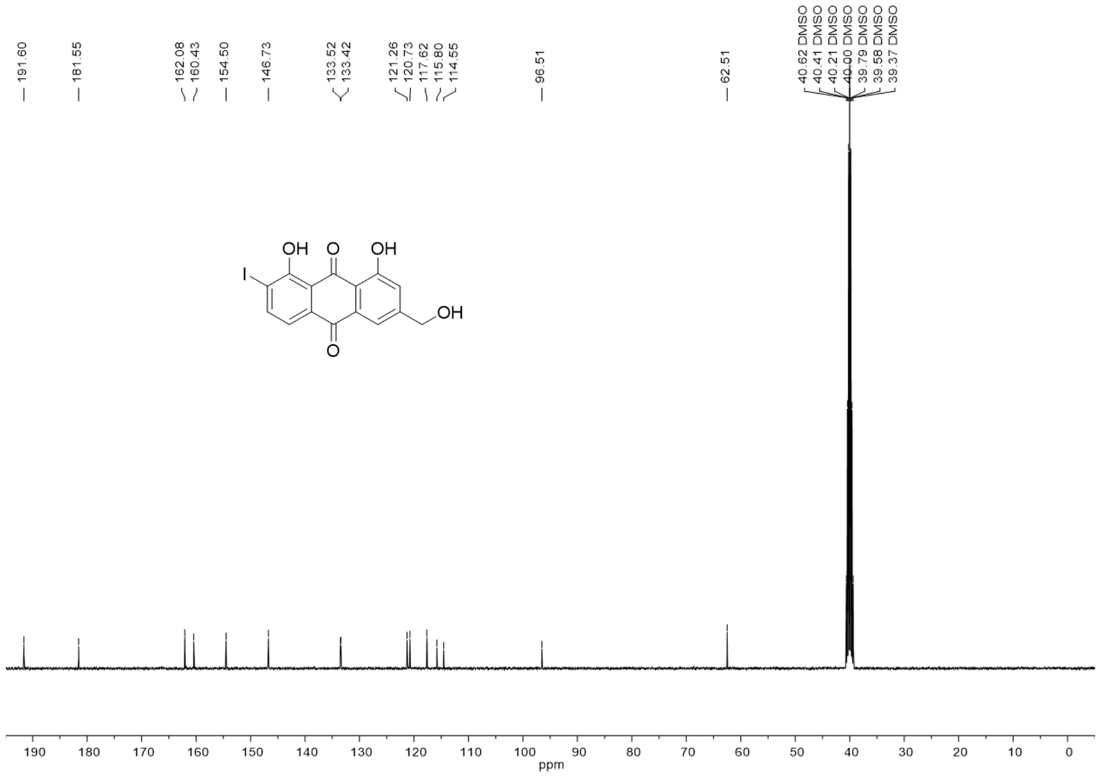

Figure S31: ^{13}C, 101 MHz, DMSO-d_6, **11**

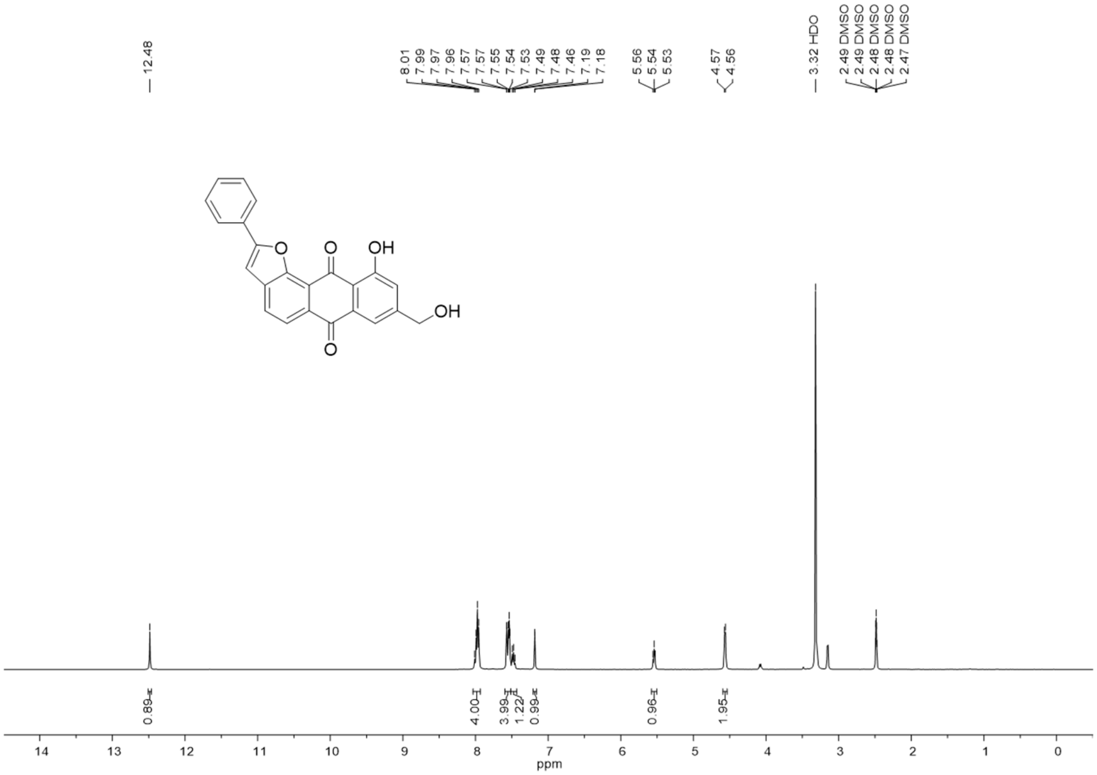

Figure S32: ^{1}H, 400 Hz, DMSO-d_{63}, **12a**

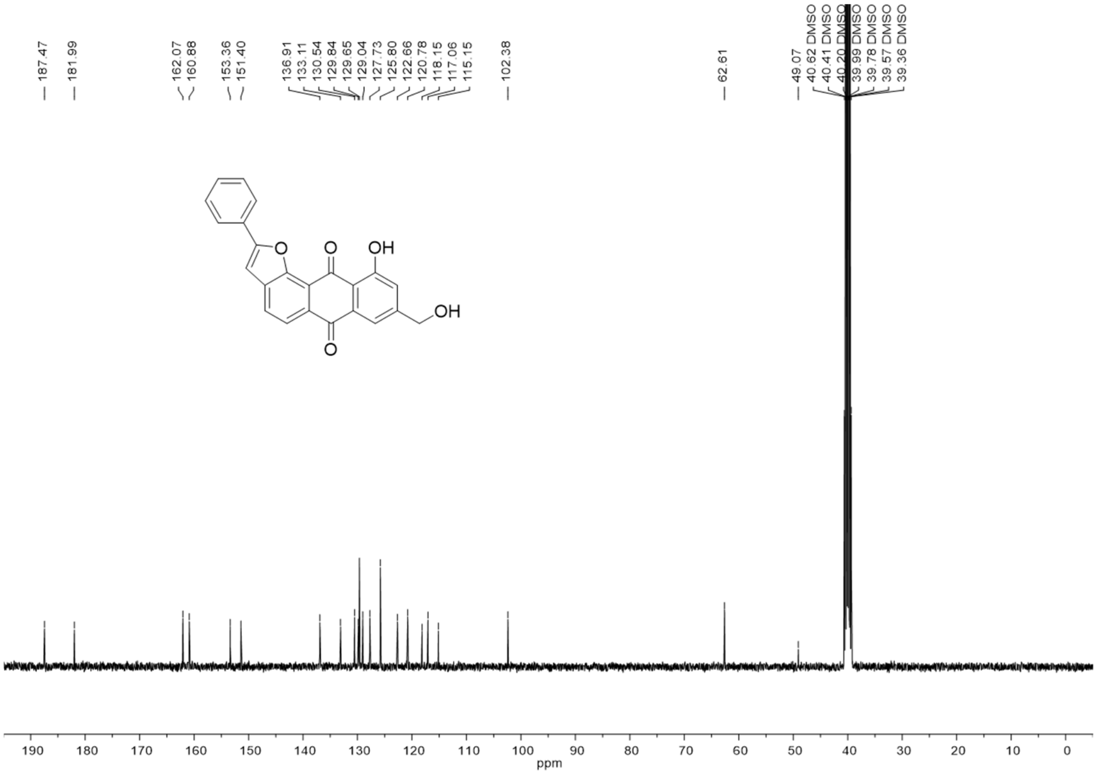

Figure S33: ^{13}C, 101 MHz, DMSO-d_6, **12a**

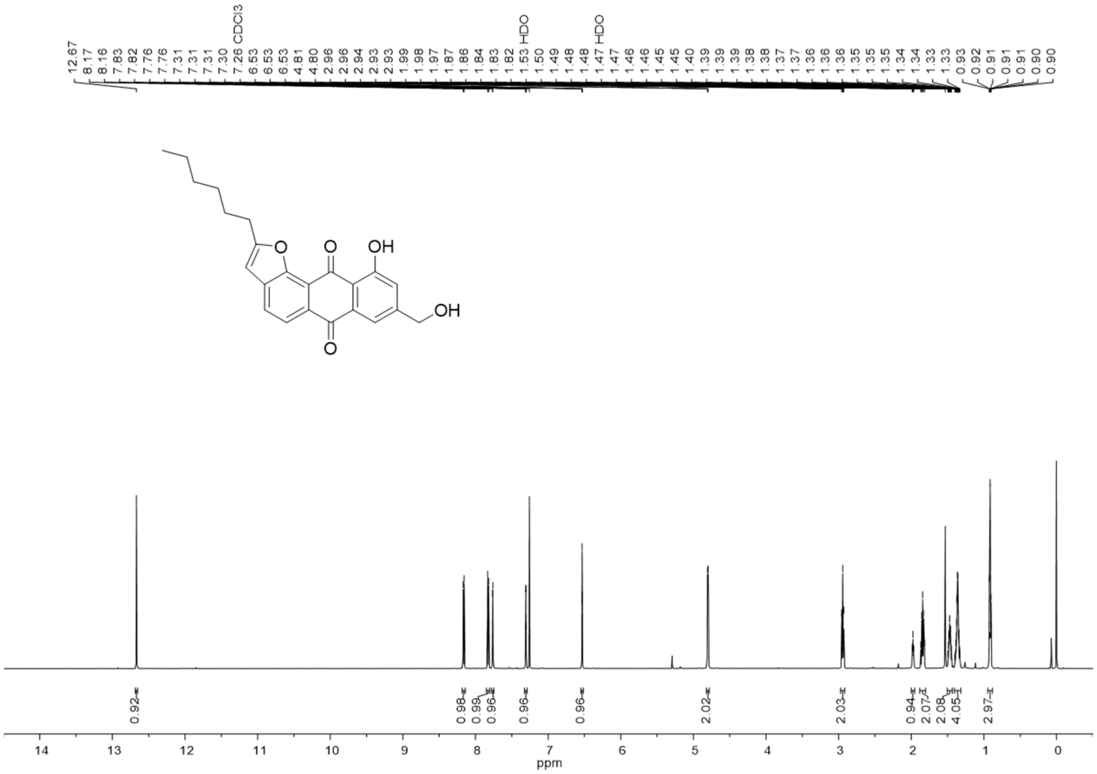

Figure S34: ^{1}H, 600 Hz, 40 ^{o}C, $CDCl_3$, **12b**

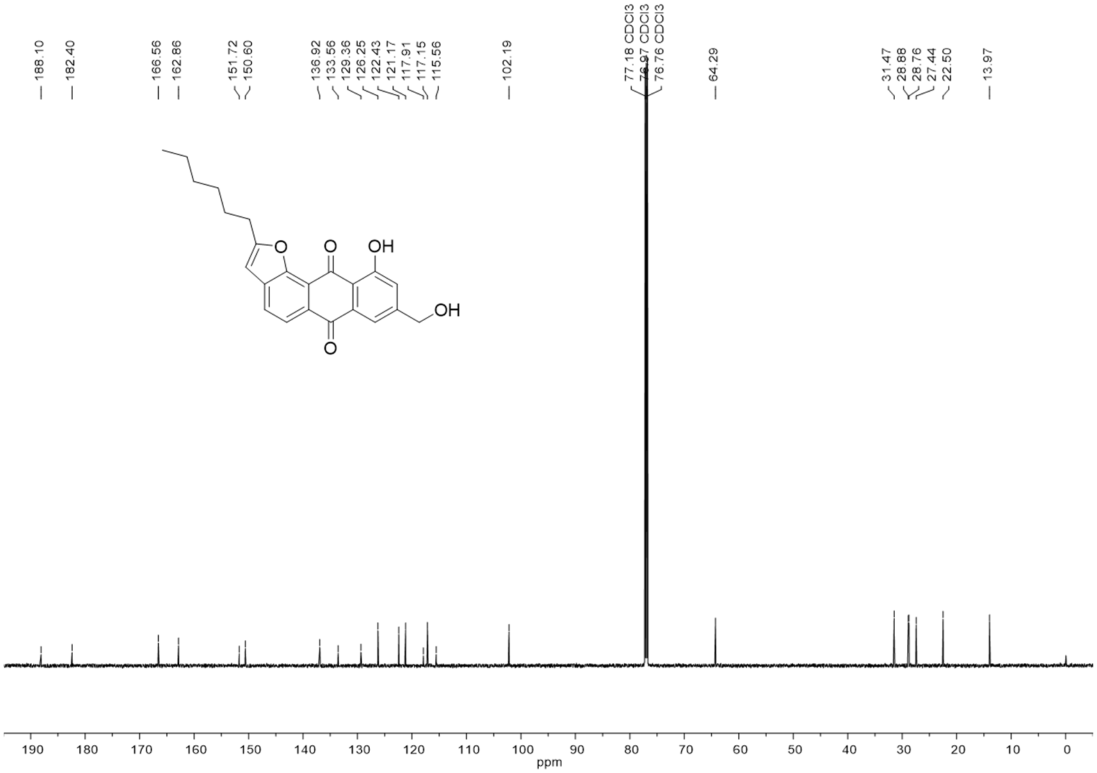

Figure S35: ^{13}C, 151 MHz, 40 ^{o}C, $CDCl_3$, **12b**

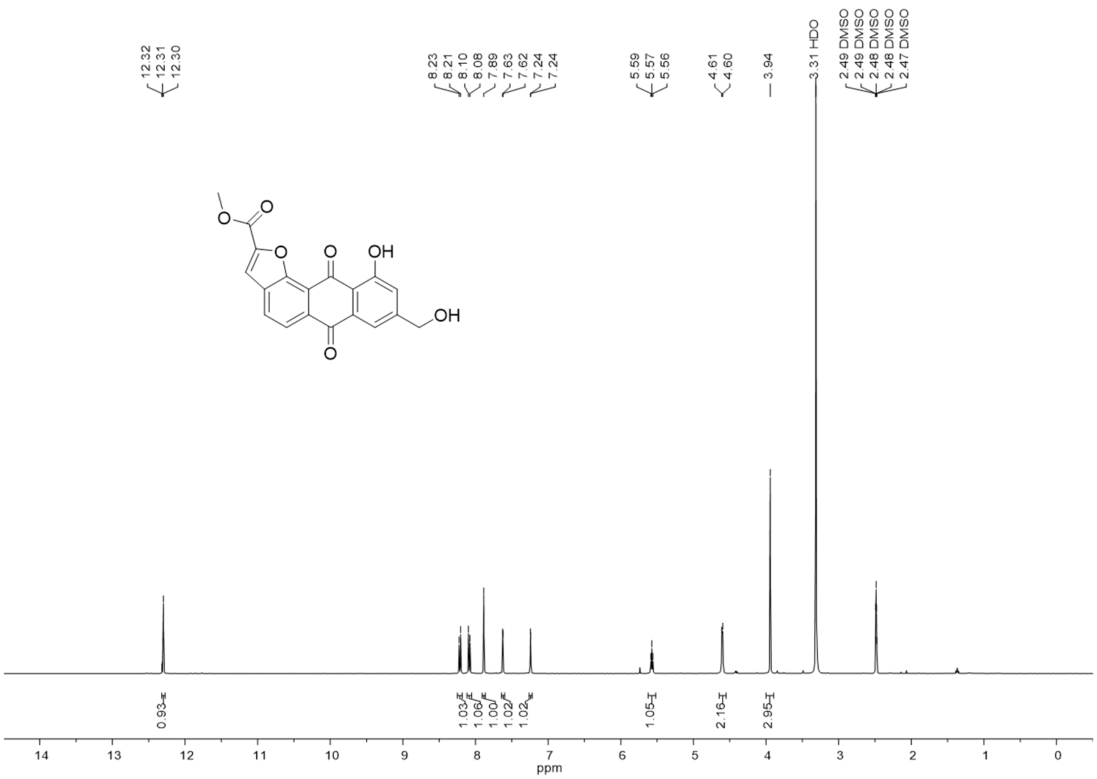

Figure S36: [1]H, 400 Hz, DMSO-d_6, **12c**

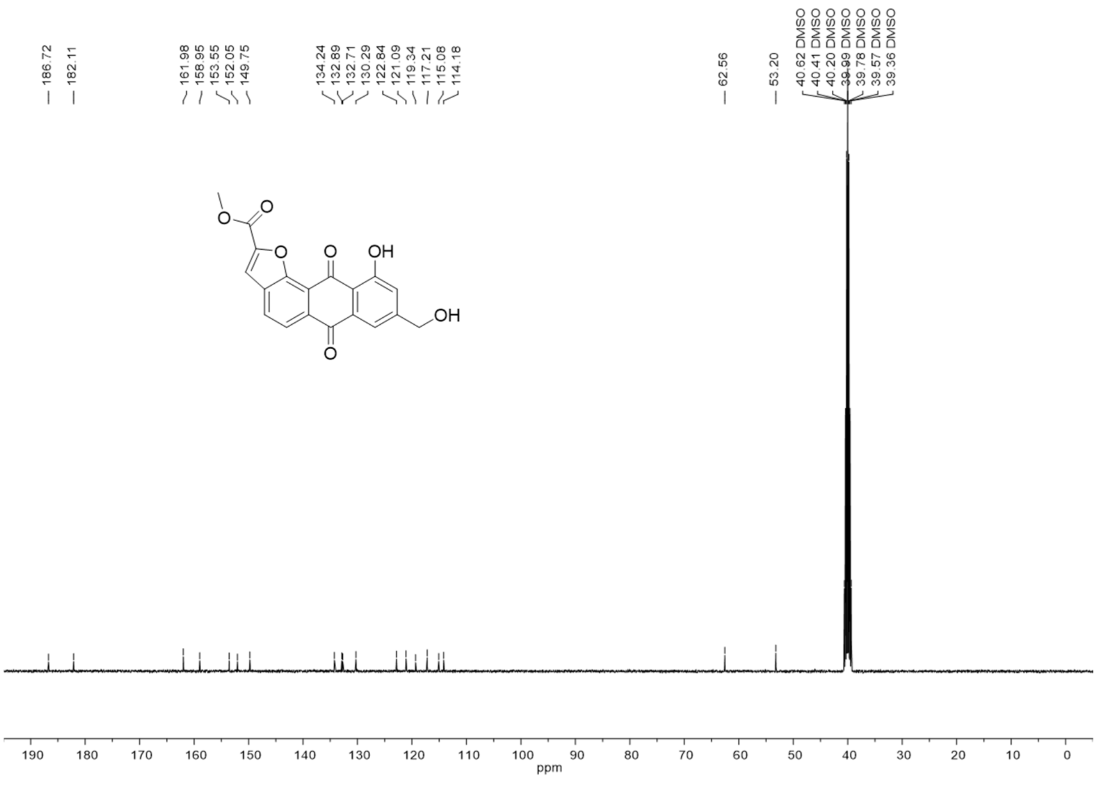

Figure S37: ^{13}C, 101 MHz, $CDCl_3$, **12c**
